# Supplementary material for: Identification of plasma proteomic markers underlying polygenic risk of type 2 diabetes and related comorbidities
Source: Nat Commun. 2025 Mar 3;16:2124. doi: 10.1038/s41467-025-56695-z (PMC11876343; doi:10.1038/s41467-025-56695-z)
Supplement: Supplementary file 1 — Supplementary Information [file 41467_2025_56695_MOESM1_ESM.pdf]

Supplementary Information for "Identification of plasma proteomic markers underlying polygenic risk of type 2 diabetes and related comorbidities"

Table of Contents

EXTENDED METHODS..2

SUPPLEMENTARY INFORMATION REFERENCES..5

SUPPLEMENTARY FIGURES...6

Extended Methods

Genotyping quality control and imputation

EXSCEL was genotyped using the Human Global Screening Array-MD-24 Beadchips v2.0 (GSA) and DECLARE was genotyped using the Infinium Global Screening Array-24 v3.0 GSA + Multi Disease (GSAMD). Quality control (QC) for imputation was performed on the genotype data using PLINK v2.00a4LM[1], removing samples with high heterozygosity (> 4 SD from the mean), discordance between reported and predicted sex, or a genotyping missingness rate > 0.05. Variants were removed if they had a HWE p-value < $1x10^{-12}$, a missingness rate > 0.05, or a minor allele frequency (MAF) < 0.001. Ancestry and relationship inference was conducted using KING 2.3.0[2] and 1000 Genomes Project data (1KGP) as an ancestral reference panel.[3,4] Subjects were assigned to a 1KGP super-population based on a genetic ancestry probability cut-off of >= 0.90. Unless otherwise stated, relative pairs were resolved to the second-degree using KING-generated kinship coefficients. Imputation in EXSCEL and DECLARE was then conducted using BEAGLE 4.0[5] and the high coverage 1KGP data as the imputation reference panel in a pipeline modelled after the FinnGen imputation pipeline.[4,6] UKB genotyping and imputation has been previously described.[7] For the estimation of polygenic scores in EXSCEL, DECLARE, and UKB, imputed variants were excluded if they had a HWE p-value < $1x10^{-6}$ within a given ancestry, a MAF < 0.01, a missingness > 0.05, and an imputation INFO score < 0.5. Relationship inference in UKB was also conducted using KING and ancestry inference was previously performed using PEDDY 0.4.2[8] with the 1KGP as the ancestral reference panel. KING and PEDDY methods for ancestry inferences are highly similar, both using 1KGP-based principal components and a SVM classifier, resulting in consistent ancestry classification as confirmed on a subset of UKB participants.

Proteomics quality control

The UKB PPP proteomics data were generated using the Olink Explore platform. Quality control was performed by the UKB-PPP consortium and is described in Sun et al. 2022.[9] For EXSCEL, proteomics data were generated for 2,949 genotyped individuals using the SomaScan assay at baseline and the 12-month timepoint. DECLARE-TIMI 58 proteomics data were generated for 934 genotyped individuals at the baseline and the 6-month timepoints using the Olink Target 96 Cardiovascular II, Cardiovascular III, Cardiometabolic panels. Proteomics quality control for the UKB was performed by the UKB-PPP consortium.[9] We followed their protocol to standardise quality control in EXSCEL and DECLARE. Samples were removed if their median expression level, IQR, or standardized PCs 1 and 2 of the corresponding proteome data were greater than 5 SD from the mean. Samples were also removed if there were any associated assay or quality control warnings issued by the company generating the proteomics data. For DECLARE, the Olink-generated normalized protein expression (npx) levels were rank-based inverse normalized. Note that covariates were not regressed from the npx values. For EXSCEL, the SomaScan data were processed using an approach modelled after Sun et al. 2018[10], i.e., protein expression data were first converted to the log10 scale, followed by regression of age, sex, PCs 1-10, and time from sample collection, and finally rank-based inverse normalized.

PGS-protein associations in randomized controlled trials

We tested all available proteins for their associations with the PGS in EXSCEL and DECLARE, adjusting for age, age^2, sex, age*sex, age^2*sex, time from sample collection (if available),

batch (if available), PCs1-10, and (+/-) BMI using the protein expression data measured at baseline prior to randomization. As a sensitivity analysis, we first included all study participants in the analyses followed by restricting them to only inferred European-ancestry participants. We applied an FDR correction to the resultant p-values.

Association of PGS with outcomes in randomized controlled trials

We tested PGS for their association with the time to EXSCEL and DECLARE outcomes with the survival package in R 3.6.1 (https://github.com/therneau/survival). We used cox proportional hazards regression and adjusted for age, sex, age*sex, PCs1-10, trial arm, and (+/-) BMI.

Supplementary Information References

1. Chang, C. C. *et al.* Second-generation PLINK: rising to the challenge of larger and richer datasets. *GigaScience* **4**, 7 (2015).
2. Manichaikul, A. *et al.* Robust relationship inference in genome-wide association studies. *Bioinformatics* **26**, 2867–2873 (2010).
3. 1000 Genomes Project Consortium *et al.* A global reference for human genetic variation. *Nature* **526**, 68–74 (2015).
4. Byrska-Bishop, M. *et al.* High-coverage whole-genome sequencing of the expanded 1000 Genomes Project cohort including 602 trios. *Cell* **185**, 3426-3440.e19 (2022).
5. Browning, B. L. & Browning, S. R. Genotype Imputation with Millions of Reference Samples. *Am. J. Hum. Genet.* **98**, 116–126 (2016).
6. Peltonen, L., Jalanko, A. & Varilo, T. Molecular genetics of the Finnish disease heritage. *Hum. Mol. Genet.* **8**, 1913–1923 (1999).
7. Bycroft, C. *et al.* The UK Biobank resource with deep phenotyping and genomic data. *Nature* **562**, 203–209 (2018).
8. Pedersen, B. S. & Quinlan, A. R. Who's Who? Detecting and Resolving Sample Anomalies in Human DNA Sequencing Studies with Peddy. *Am. J. Hum. Genet.* **100**, 406–413 (2017).
9. Sun, B. B. *et al.* Genetic regulation of the human plasma proteome in 54,306 UK Biobank participants. 2022.06.17.496443 Preprint at https://doi.org/10.1101/2022.06.17.496443 (2022).
10. Sun, B. B. *et al.* Genomic atlas of the human plasma proteome. *Nature* **558**, 73–79 (2018).

09/02/2025 17:04:00Supplementary Figures

A.

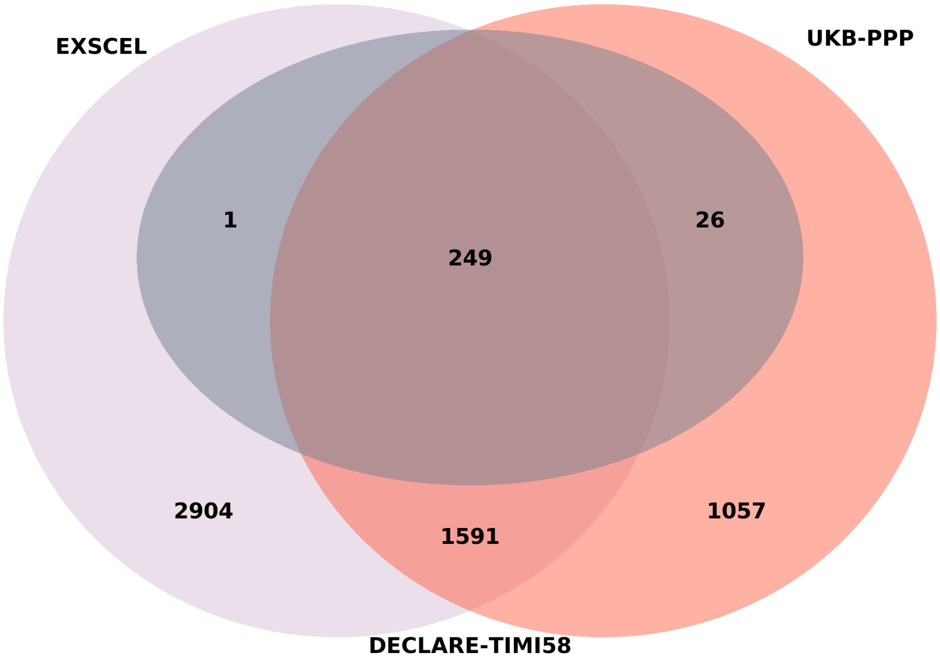

B.

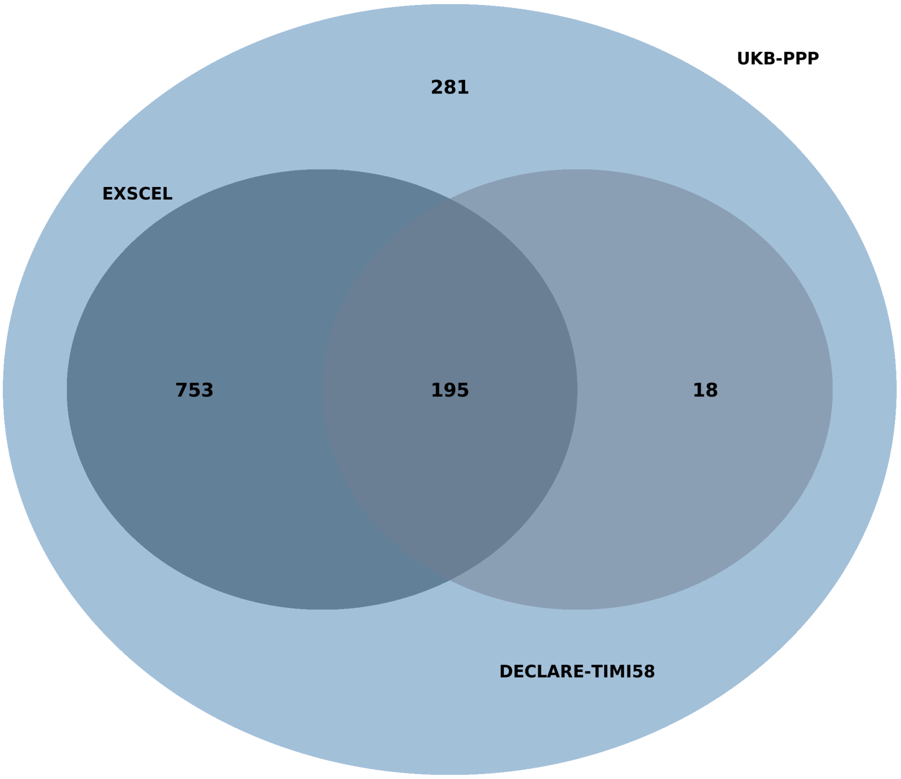

Supplementary Figure 1: Proteomics overlap. A: Venn diagram of protein intersections across UKB, EXSCEL, and DECLARE-TIMI58. The purple circle corresponds to EXSCEL, the red circle corresponds to UKB-PPP, and the grey circle (completely internal to the other sets) corresponds to DECLARE-TIMI58. B: Venn diagram of PGS-significant protein intersections across UKB, EXSCEL, and DECLARE-TIMI58. Note that restricts proteins to only those available in UKB. The large blue circle are PGS-significant proteins in UKB, the dark blue circle within the UKB circle corresponds to EXSCEL, and the grey circle to the right corresponds to DECLARE.

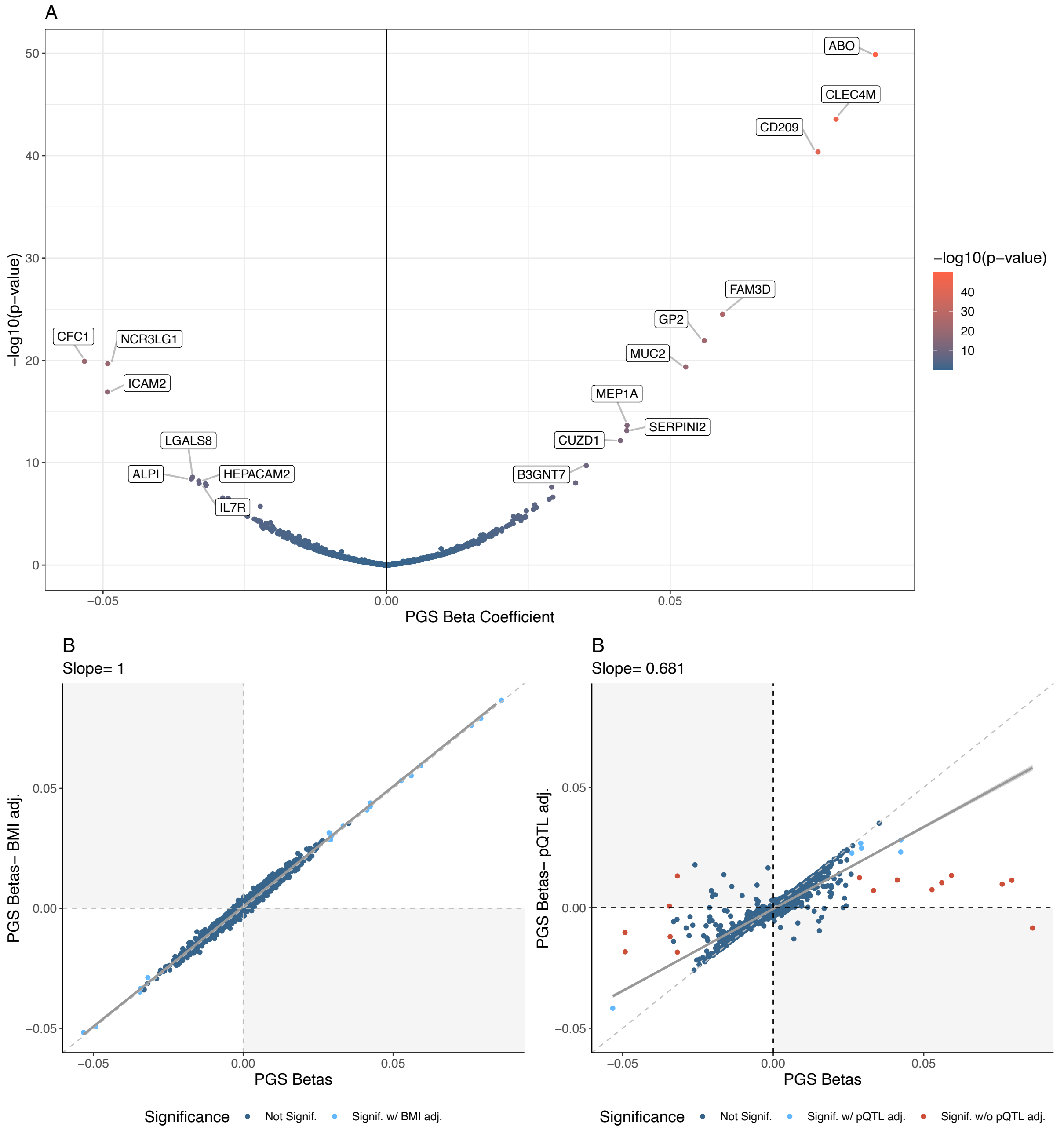

Supplementary Figure 2: A: Volcano plot of $PGS_{T2D\_beta\_cell}$ associations using linear regression and 29,496 UKB participants from the Discovery subset (see Methods). Labelled proteins were the top 0.5% of $PGS_{T2D\_beta\_cell}$ associations by R^2. B: Beta-beta plot of pPS-protein associations with (Y-axis) and without BMI-adjustment (X-axis). The regression line is solid grey while the diagonal is dashed grey. C. Beta-beta plot of pPS-protein associations with (Y-axis) and without pQTL-adjustment (X-axis). The regression line is solid grey while the diagonal is dashed grey. See Figure 1 for a description of the colouring schemes for panels A-C.

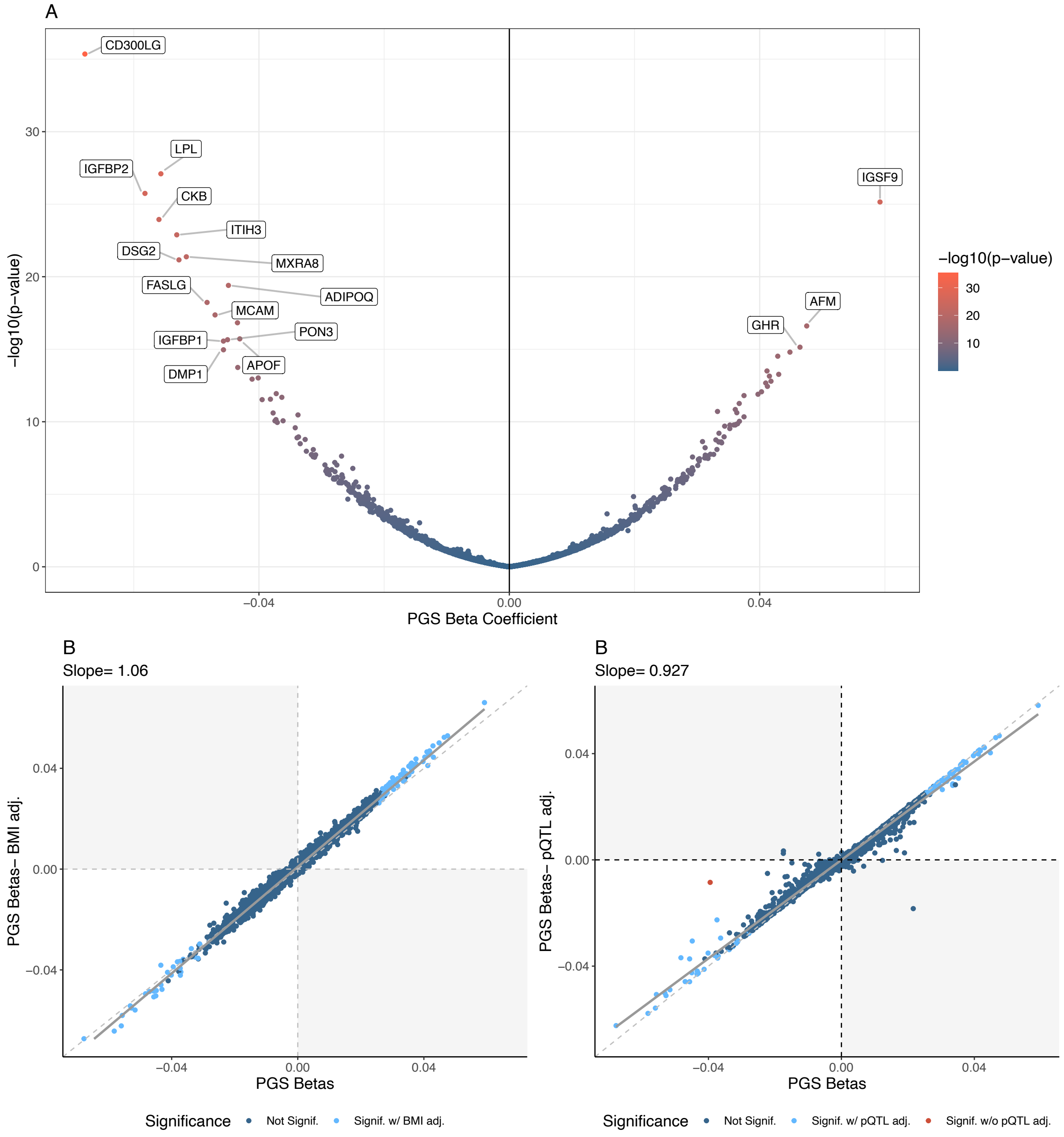

Supplementary Figure 3: A: Volcano plot of $PGS_{T2D\_lipodystrophy}$ associations using linear regression and 29,496 UKB participants from the Discovery subset (see Methods). Labelled proteins were the top 0.5% of $PGS_{T2D\_lipodystrophy}$ associations by R^2. B: Beta-beta plot of pPS-protein associations with (Y-axis) and without BMI-adjustment (X-axis). The regression line is solid grey while the diagonal is dashed grey. C. Beta-beta plot of pPS-protein associations with (Y-axis) and without pQTL-adjustment (X-axis). The regression line is solid grey while the diagonal is dashed grey. See Figure 1 for a description of the colouring schemes for panels A-C.

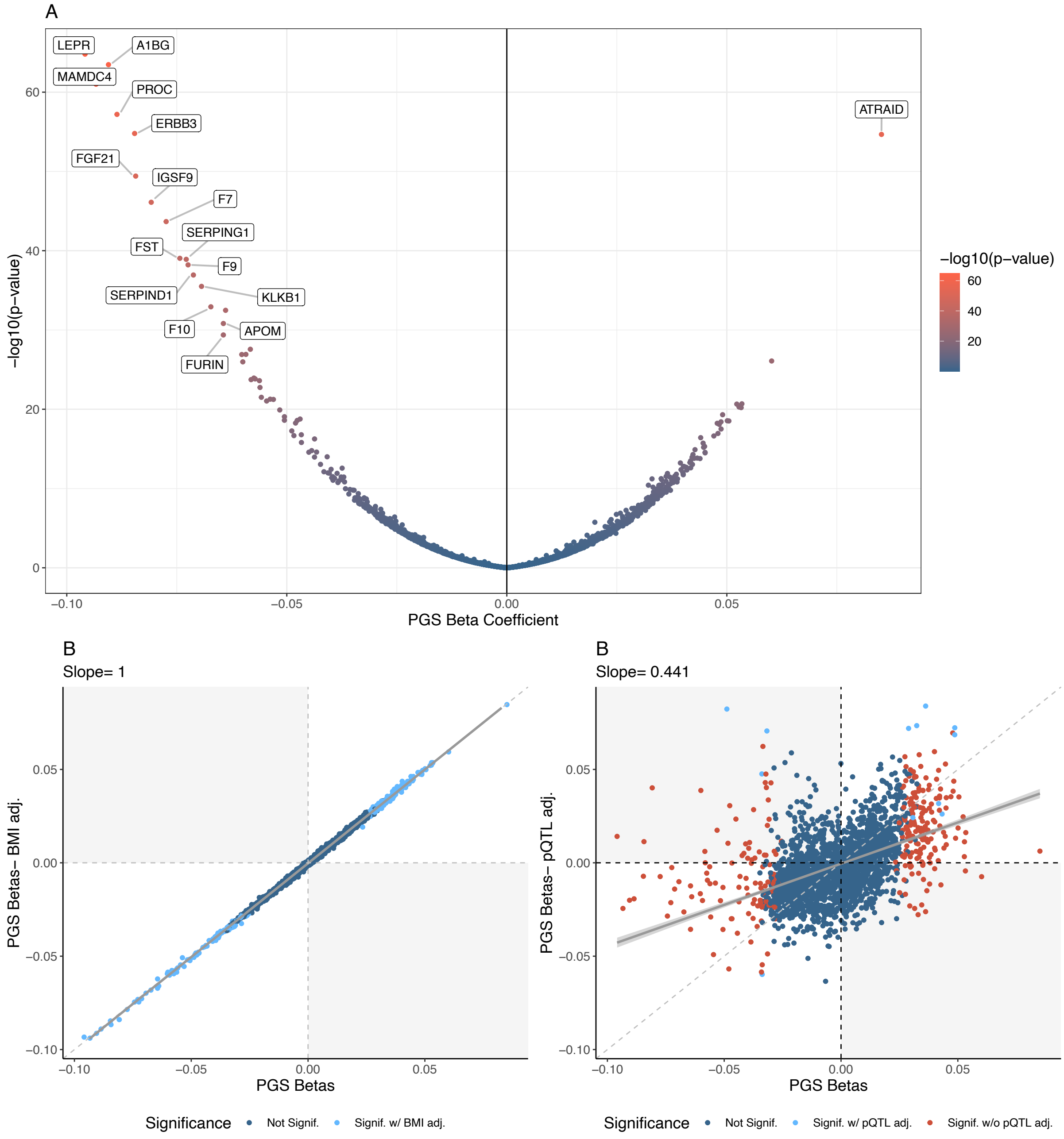

Supplementary Figure 4: A: Volcano plot of $PGS_{T2D\_liver\_lipid}$ associations using linear regression and 29,496 UKB participants from the Discovery subset (see Methods). Labelled proteins were the top 0.5% of $PGS_{T2D\_liver\_lipid}$ associations by R^2. B: Beta-beta plot of pPS-protein associations with (Y-axis) and without BMI-adjustment (X-axis). The regression line is solid grey while the diagonal is dashed grey. C. Beta-beta plot of pPS-protein associations with (Y-axis) and without pQTL-adjustment (X-axis). The regression line is solid grey while the diagonal is dashed grey. See Figure 1 for a description of the colouring schemes for panels A-C.

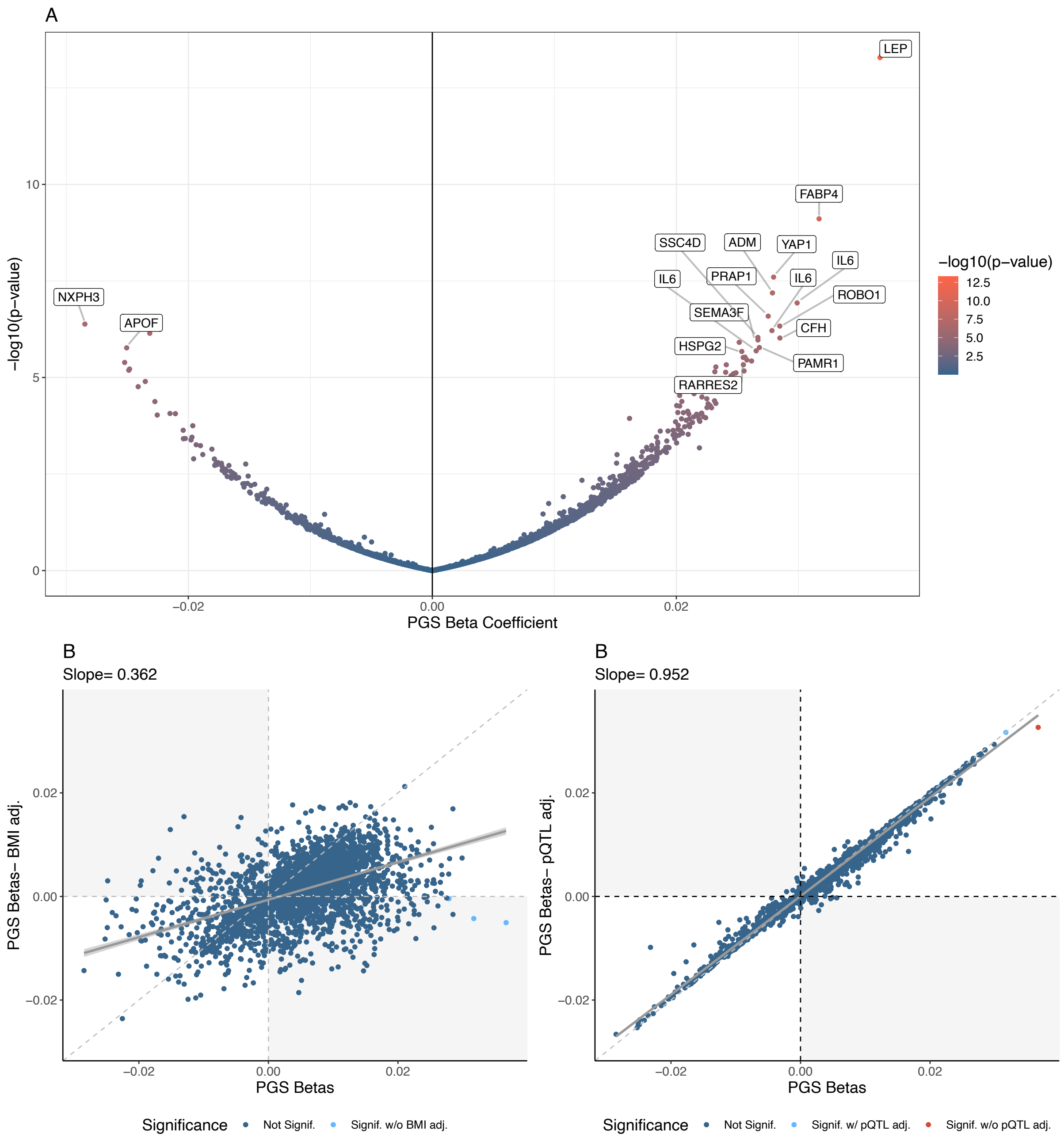

Supplementary Figure 5: A: Volcano plot of $PGS_{T2D\_obesity}$ associations using linear regression and 29,496 UKB participants from the Discovery subset (see Methods). Labelled proteins were the top 0.5% of $PGS_{T2D\_obesity}$ associations by R^2. B: Beta-beta plot of pPS-protein associations with (Y-axis) and without BMI-adjustment (X-axis). The regression line is solid grey while the diagonal is dashed grey. C. Beta-beta plot of pPS-protein associations with (Y-axis) and without pQTL-adjustment (X-axis). The regression line is solid grey while the diagonal is dashed grey. See Figure 1 for a description of the colouring schemes for panels A-C.

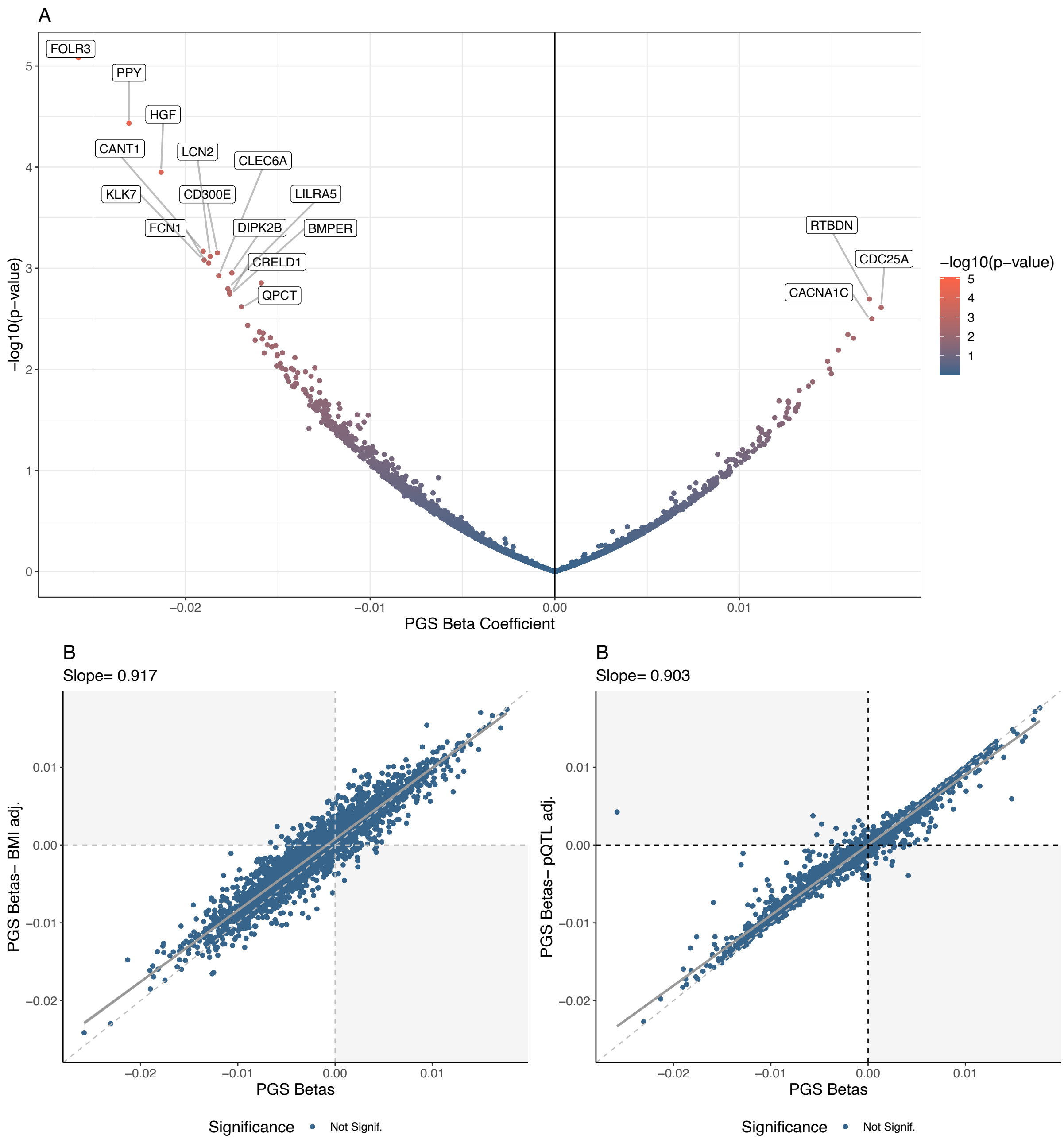

Supplementary Figure 6: A: Volcano plot of $PGS_{T2D\_proinsulin}$ associations using linear regression and 29,496 UKB participants from the Discovery subset (see Methods). Labelled proteins were the top 0.5% of $PGS_{T2D\_proinsulin}$ associations by R^2. B: Beta-beta plot of pPS-protein associations with (Y-axis) and without BMI-adjustment (X-axis). The regression line is solid grey while the diagonal is dashed grey. C. Beta-beta plot of pPS-protein associations with (Y-axis) and without pQTL-adjustment (X-axis). The regression line is solid grey while the diagonal is dashed grey. See Figure 1 for a description of the colouring schemes for panels A-C.

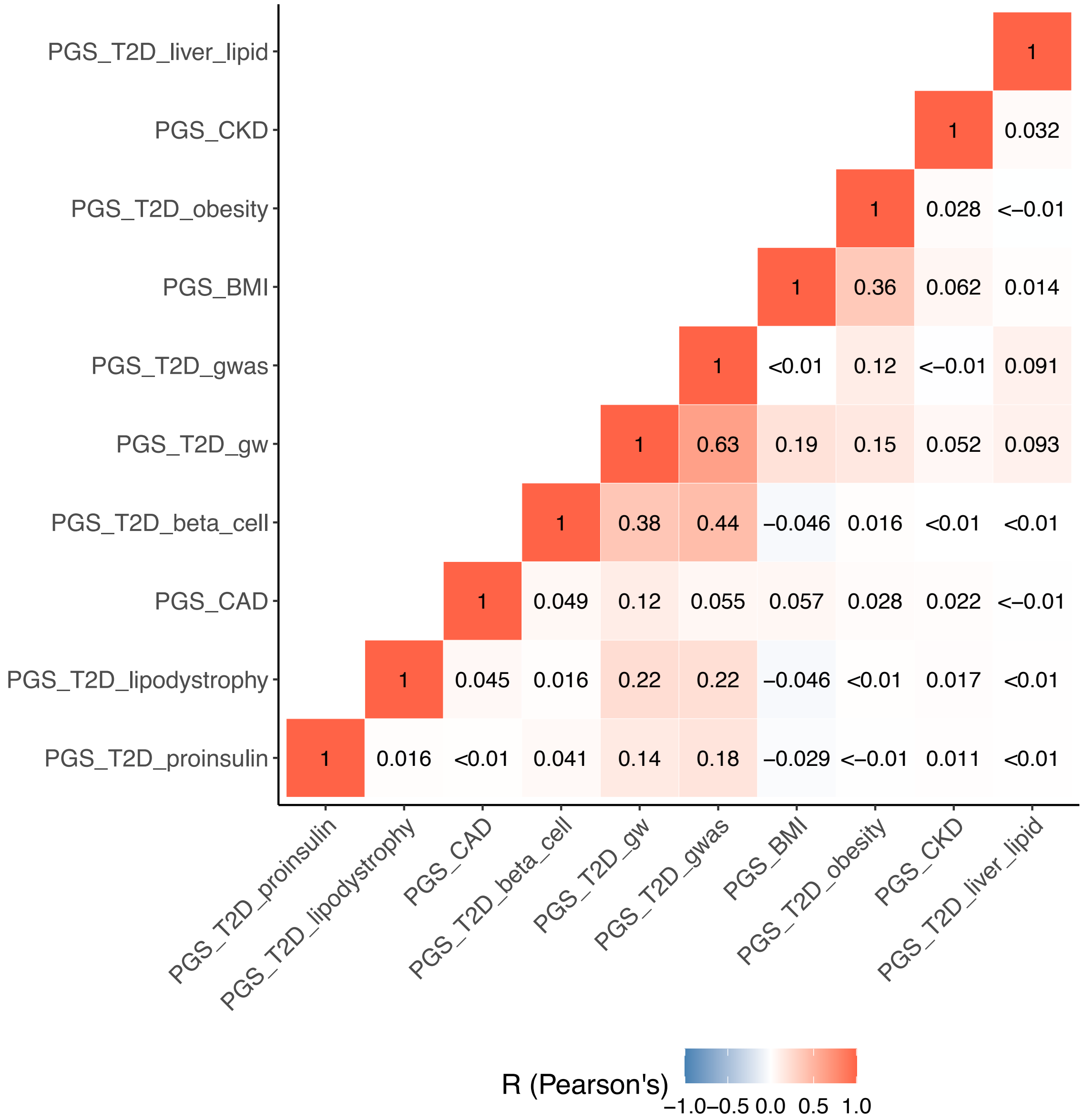

Supplementary Figure 7: Correlation of Polygenic Scores (PGS) in the UKB-PPP cohort. Heatmap of correlations (Pearson's R) between the PGS (Pearson's R) evaluated in this study using the unrelated subset of the UKB-PPP cohort (N=44,381).

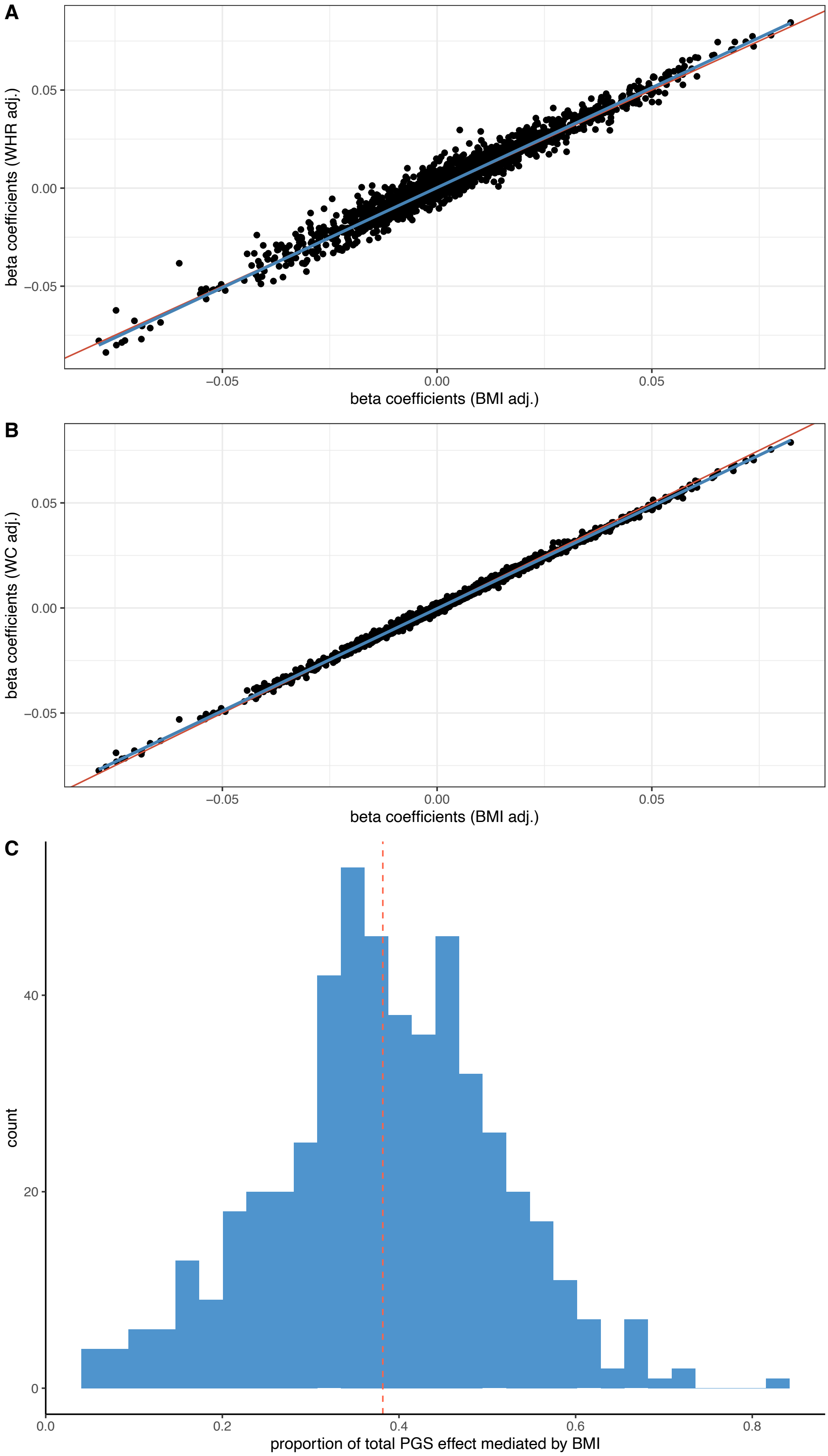
A
beta coefficients (WHR adj.)
0.05
0.00
−0.05
−0.05
0.00
0.05
beta coefficients (BMI adj.)
B
beta coefficients (WC adj.)
0.05
0.00
−0.05
−0.05
0.00
0.05
beta coefficients (BMI adj.)
C
count
40
20
0
0.0
0.2
0.4
0.6
0.8
proportion of total PGS effect mediated by BMI

Supplementary Figure 8: Adiposity, T2D PGS, and circulating proteins. A. Beta-beta plot showing beta coefficients of the PGS_{T2D_gw} in a regression (N= 44,381) on circulating proteins after adjusting for body mass index (BMI, on the x-axis) and after adjusting for waist-hip ratio (WHR, y-axis). B. Beta-beta plot showing beta coefficients of the PGS_{T2D_gw} in a regression on circulating proteins after adjusting for BMI (x-axis) and after adjusting for waist circumference ratio (WC, y-axis). All adiposity measurements were taken at baseline. C. Histogram showing the distribution of the proportion of the PGS_{T2D_gw} that was mediated by BMI. Mediation analysis was done using the mediation R package and a sample size of 44,381 UK Biobank participants.

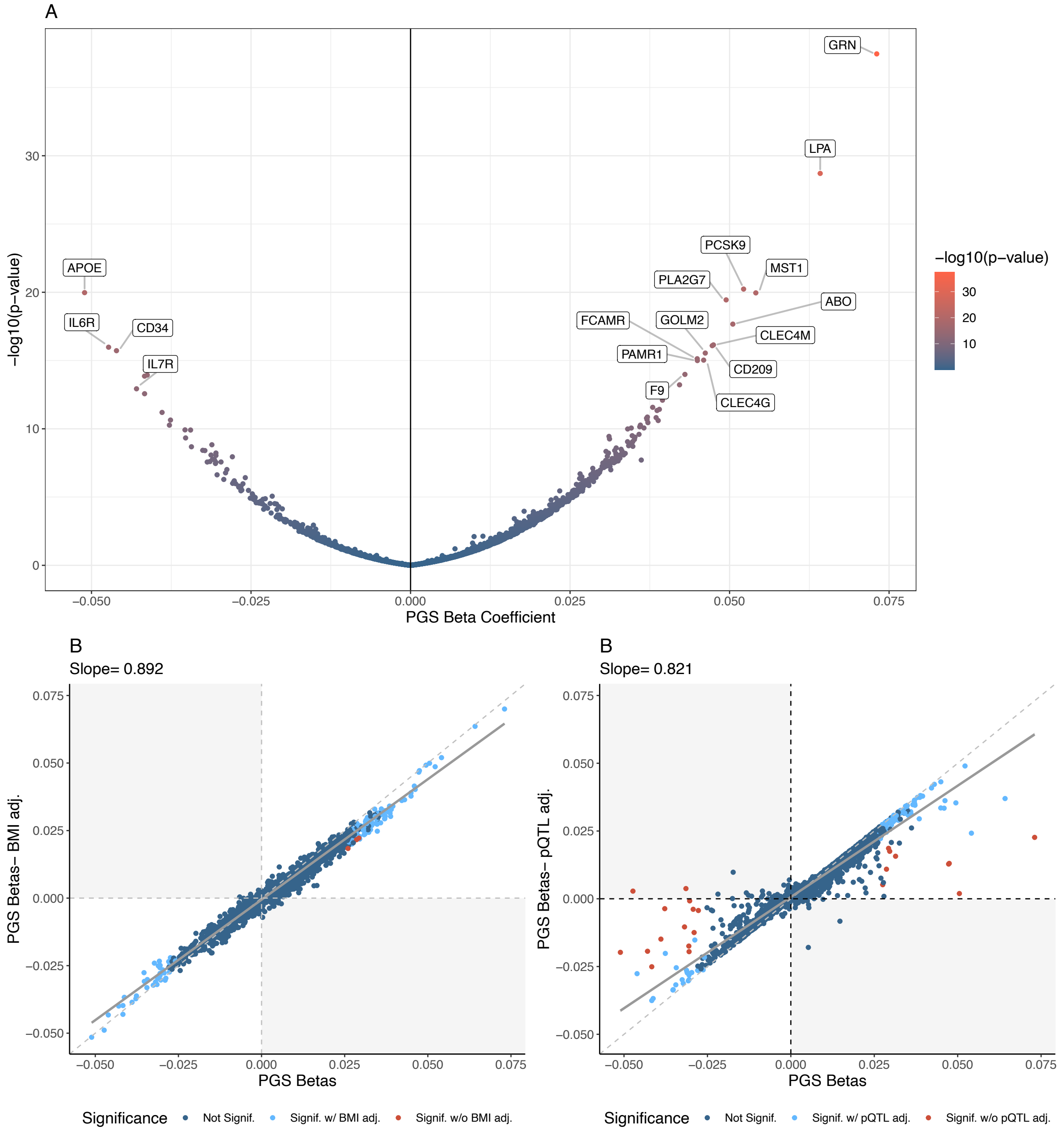

Supplementary Figure 9: PGS_{CAD} plot. A: Volcano plot of PGS_{CAD} associations using linear regression and 29,496 UKB participants from the Discovery subset (see Methods). Labelled proteins were the top 0.5% of PGS associations by R^2. B: Beta-beta plot of PGS-protein associations with (Y-axis) and without BMI-adjustment (X-axis). The regression line is solid grey while the diagonal is dashed grey. C. Beta-beta plot of PGS-protein associations with (Y-axis) and without pQTL-adjustment (X-axis). The regression line is solid grey while the diagonal is dashed grey. See Figure 1 for a description of the colouring schemes for panels A-C.

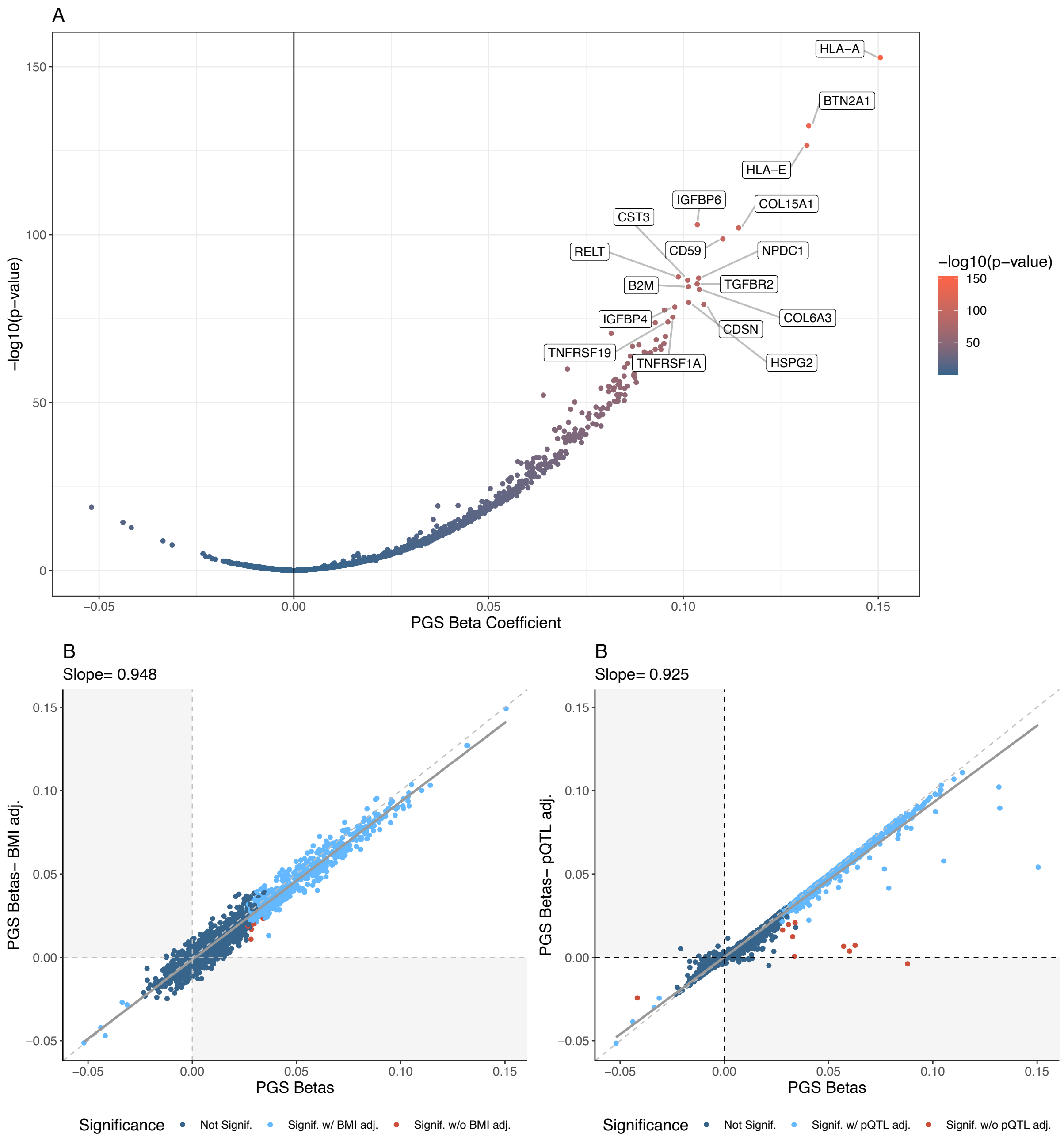

Supplementary Figure 10: PGS_{CKD} plot. A: Volcano plot of PGS_{CKD} associations using linear regression and 29,496 UKB participants from the Discovery subset (see Methods). Labelled proteins were the top 0.5% of PGS associations by R^2. B: Beta-beta plot of PGS-protein associations with (Y-axis) and without BMI-adjustment (X-axis). The regression line is solid grey while the diagonal is dashed grey. C. Beta-beta plot of PGS-protein associations with (Y-axis) and without pQTL-adjustment (X-axis). The regression line is solid grey while the diagonal is dashed grey. See Figure 1 for a description of the colouring schemes for panels A-C.

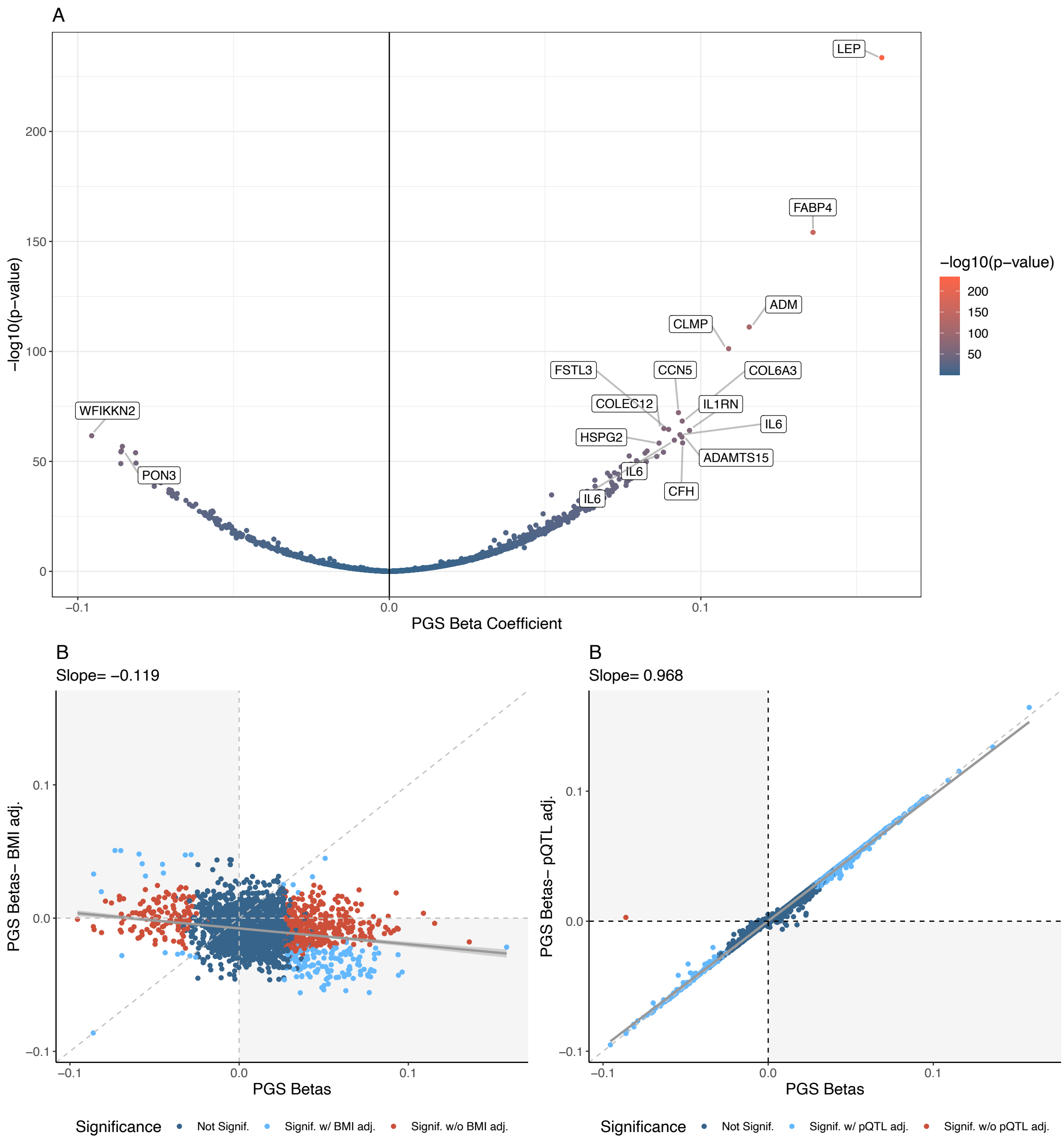

Supplementary Figure 11: PGS$_{BMI}$ plot. A: Volcano plot of PGS$_{BMI}$ associations. Labelled proteins were the top 0.5% of PGS associations by R^2. B: Beta-beta plot of PGS-protein associations with (Y-axis) and without BMI-adjustment (X-axis). The regression line is solid grey while the diagonal is dashed grey. C. Beta-beta plot of PGS-protein associations with (Y-axis) and without pQTL-adjustment (X-axis). The regression line is solid grey while the diagonal is dashed grey. See Figure 1 for a description of the colouring schemes for panels A-C.

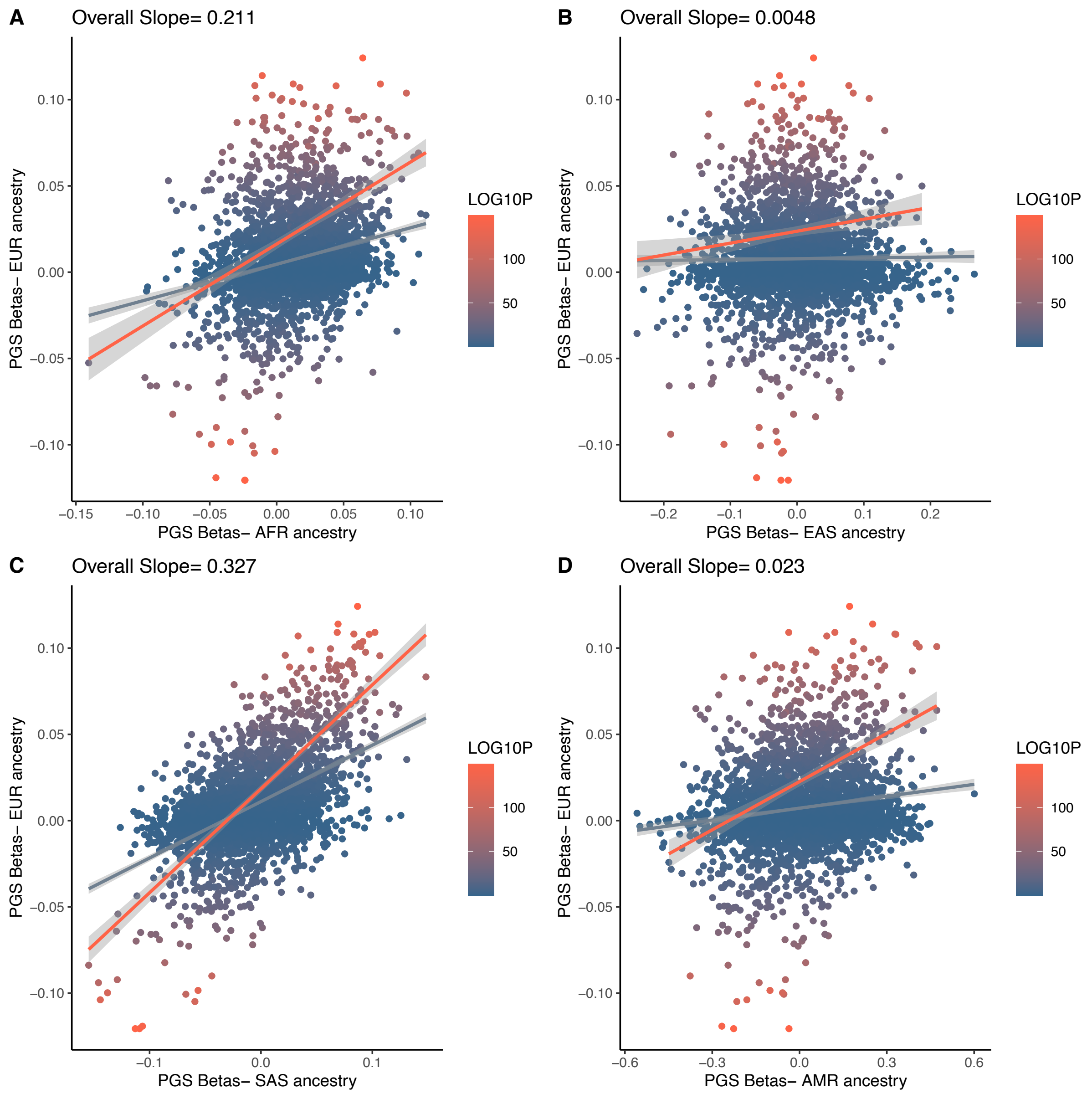

Supplementary Figure 12: Cross-population comparison of PGS_{T2D_gw} effect sizes on circulating protein levels. Ancestry labels were genetically predicted using the 1000 Genomes Project (1KGP) and 1KGP super-population categories. A. Beta-beta plot of PGS effect sizes in European ancestry (EUR, or 1KGP EUR-like on the y-axis; N= 41,453) and African ancestry (AFR, or 1KGP AFR-like on the x-axis; N= 1,205) UKB participants. B. Beta-beta plot of PGS effect sizes in European ancestry and East Asian ancestry (EAS, or 1KGP EAS-like on the x-axis; N= 226) UKB participants. C. Beta-beta plot of PGS effect sizes in European ancestry and South Asian ancestry (SAS, or 1KGP SAS-like on the x-axis; N= 836) UKB participants. D. Beta-beta plot of PGS effect sizes in European ancestry and Admixed American (Latin American/Hispanic) ancestry (AMR or 1KGP AMR-like on the x-axis; N=76) UKB participants. For panels A-D, the regression line for all proteins is silver and the regression line for only replicated proteins is in red. All beta coefficients were obtained via linear regression. Note that the East Asian and Latin American subsets were underpowered (B and D), likely impacting the results.

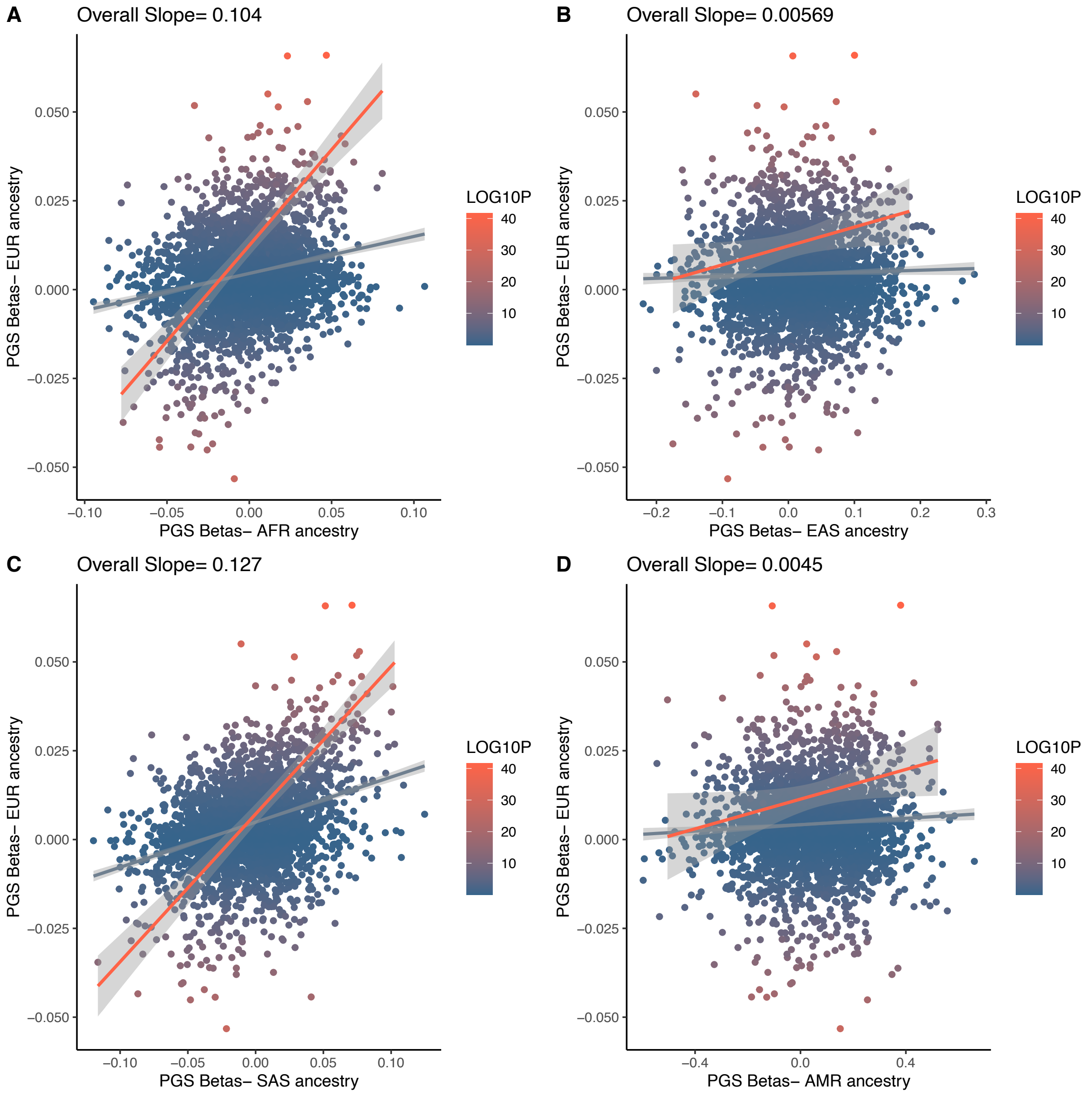

Supplementary Figure 13: Cross-population comparison of PGS_{CAD} effect sizes on circulating protein levels. Ancestry labels were genetically predicted using the 1000 Genomes Project (1KGP) and 1KGP super-population categories. A. Beta-beta plot of PGS effect sizes in European ancestry (EUR, or 1KGP EUR-like on the y-axis; N= 41,453) and African ancestry (AFR, or 1KGP AFR-like on the x-axis; N= 1,205) UKB participants. B. Beta-beta plot of PGS effect sizes in European ancestry and East Asian ancestry (EAS, or 1KGP EAS-like on the x-axis; N= 226) UKB participants. C. Beta-beta plot of PGS effect sizes in European ancestry and South Asian ancestry (SAS, or 1KGP SAS-like on the x-axis; N= 836) UKB participants. D. Beta-beta plot of PGS effect sizes in European ancestry and Admixed American (Latin American/Hispanic) ancestry (AMR or 1KGP AMR-like on the x-axis; N=76) UKB participants. For panels A-D, the regression line for all proteins is silver and the regression line for only replicated proteins is in red. All beta coefficients were obtained via linear regression. Note that the East Asian and Latin American subsets were underpowered (B and D), likely impacting the results.

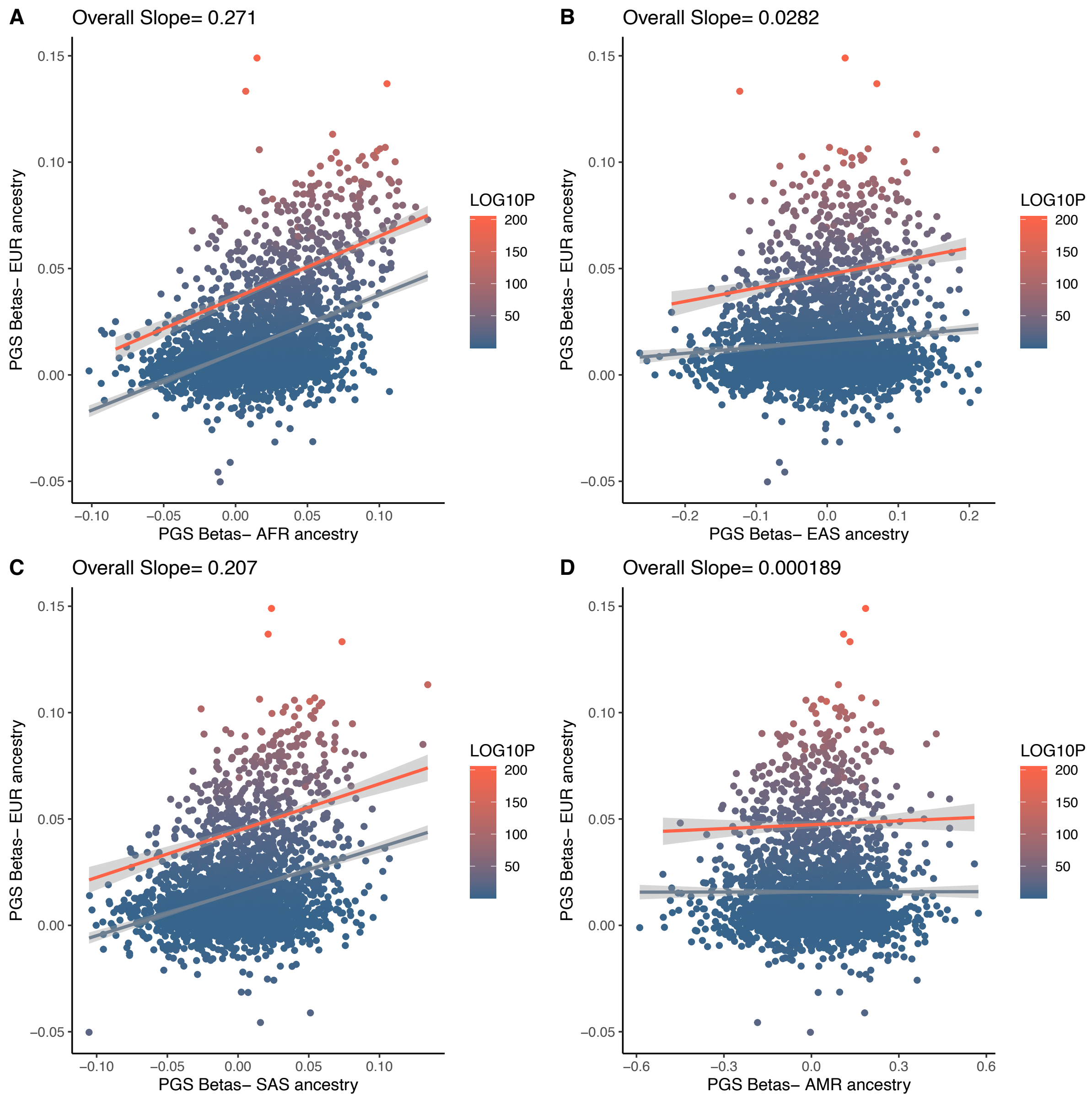

Supplementary Figure 14: Cross-population comparison of PGS_{CKD} effect sizes on circulating protein levels. Ancestry labels were genetically predicted using the 1000 Genomes Project (1KGP) and 1KGP super-population categories. A. Beta-beta plot of PGS effect sizes in European ancestry (EUR, or 1KGP EUR-like on the y-axis; N= 41,453) and African ancestry (AFR, or 1KGP AFR-like on the x-axis; N= 1,205) UKB participants. B. Beta-beta plot of PGS effect sizes in European ancestry and East Asian ancestry (EAS, or 1KGP EAS-like on the x-axis; N= 226) UKB participants. C. Beta-beta plot of PGS effect sizes in European ancestry and South Asian ancestry (SAS, or 1KGP SAS-like on the x-axis; N= 836) UKB participants. D. Beta-beta plot of PGS effect sizes in European ancestry and Admixed American (Latin American/Hispanic) ancestry (AMR or 1KGP AMR-like on the x-axis; N=76) UKB participants. For panels A-D, the regression line for all proteins is silver and the regression line for only replicated proteins is in red. All beta coefficients were obtained via linear regression. Note that the East Asian and Latin American subsets were underpowered (B and D), likely impacting the results.

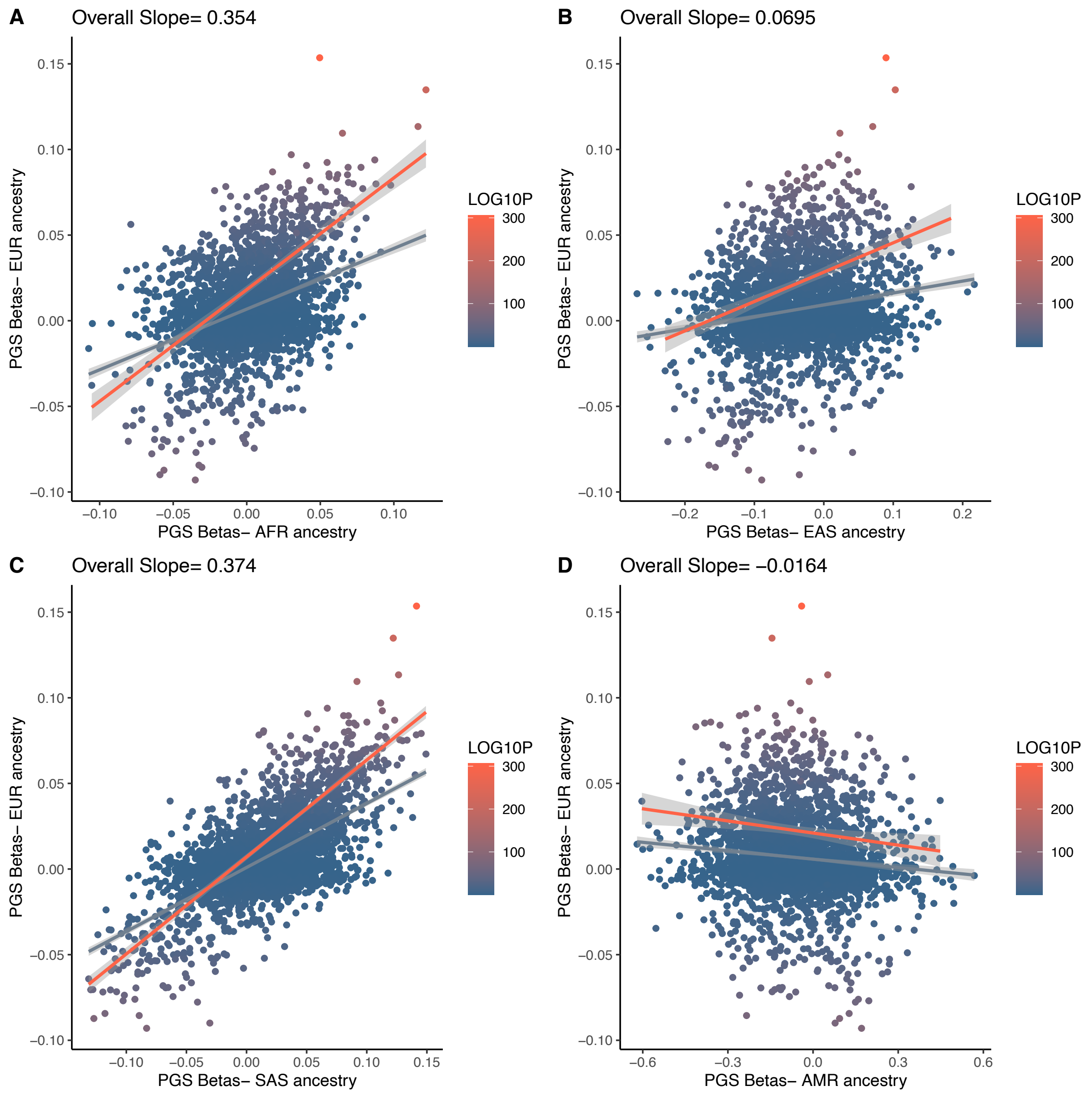

Supplementary Figure 15: Cross-population comparison of PGS_{BMI} effect sizes on circulating protein levels. Ancestry labels were genetically predicted using the 1000 Genomes Project (1KGP) and 1KGP super-population categories. A. Beta-beta plot of PGS effect sizes in European ancestry (EUR, or 1KGP EUR-like on the y-axis; N= 41,453) and African ancestry (AFR, or 1KGP AFR-like on the x-axis; N= 1,205) UKB participants. B. Beta-beta plot of PGS effect sizes in European ancestry and East Asian ancestry (EAS, or 1KGP EAS-like on the x-axis; N= 226) UKB participants. C. Beta-beta plot of PGS effect sizes in European ancestry and South Asian ancestry (SAS, or 1KGP SAS-like on the x-axis; N= 836) UKB participants. D. Beta-beta plot of PGS effect sizes in European ancestry and Admixed American (Latin American/Hispanic) ancestry (AMR or 1KGP AMR-like on the x-axis; N=76) UKB participants. For panels A-D, the regression line for all proteins is silver and the regression line for only replicated proteins is in red. All beta coefficients were obtained via linear regression. Note that the East Asian and Latin American subsets were underpowered (B and D), likely impacting the results.

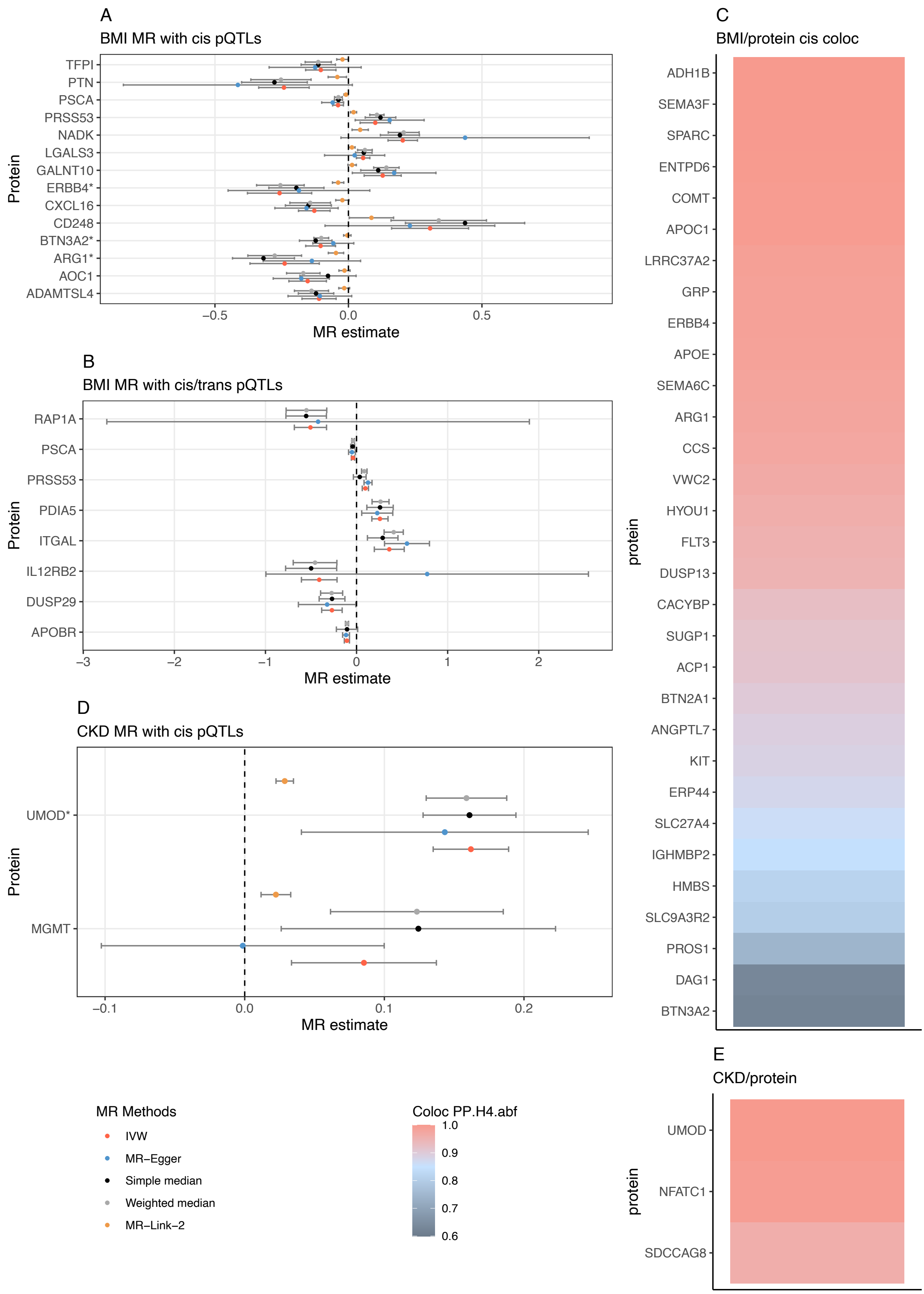

Supplementary Figure 16: MR analysis of BMI and CKD in UKB. A. Body mass index (BMI) MR with cis instruments for each protein as the exposure. B. BMI MR with both cis and trans instruments for each protein as the exposure. C. Cis colocalization using BMI and pQTL GWAS information. D. Chronic kidney disease (CKD) MR using cis instruments for each

protein as the exposure. E. CKD colocalization using CKD and pQTL GWAS information. In the MR plots, four conventional MR methods are displayed (inverse variance weighted, simple median, weighted median, and Egger), plus an additional MR method called MR-Link-2. Note that MR-Link-2 estimates are on a different scale than the other MR methods but shows consistency of effect. For proteins to be included in this plot, they needed to have median p-value across the four conventional MR methods with an FDR corrected p-value < 0.05 and no pleiotropy as detected by MR-Egger (MR-Egger intercept p-value > 0.05). MR estimates were obtained from the MendelianRanomization R package and MR-Link-2. Error bars represent the 95% confidence interval using the standard error of each MR estimate. Finally, "*" signifies proteins with colocalization evidence.

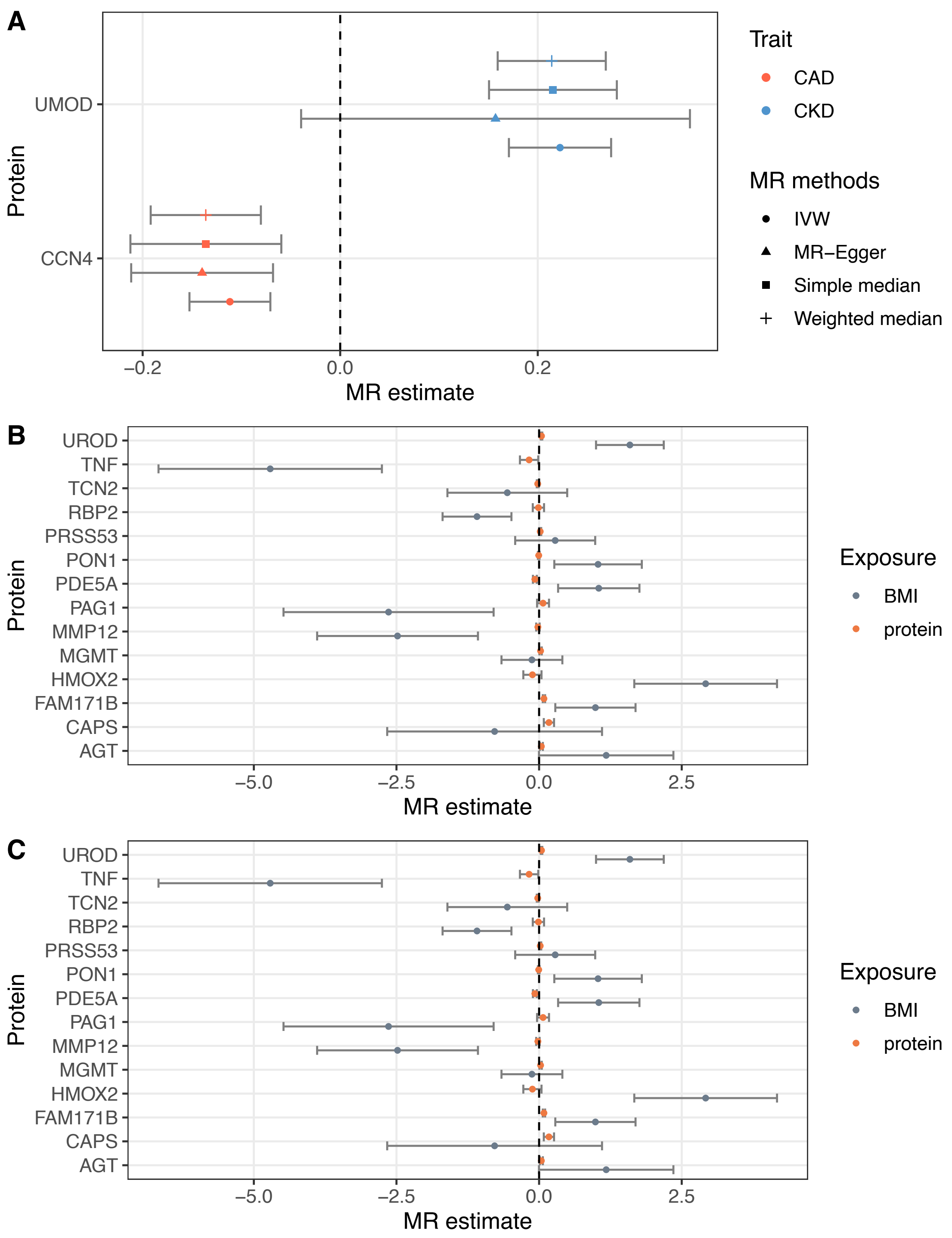

Supplementary Figure 17: multivariable Mendelian randomization (MR) analysis and MR of CVD/CKD in UKB patients with T2D. A. MR analysis of cardiorenal outcomes in UK Biobank participants with T2D using *cis* instruments. B. *Cis* multivariable MR using body mass index (BMI) instruments (from FinnGen) and protein instruments (from UK Biobank) as exposures and T2D GWAS from the UK Biobank (excluding participants in the proteomics cohort). In this plot, the F-statistic for both exposures were above 10, without evidence of pleiotropy (Q-statistic p-value > 0.05), and one of the exposures had a nominally significant MR estimate **(**p-value < 0.05). C. Same analysis as in panel B, but in this case uses both *cis* and *trans* instruments. For all three panels, error bars represent the 95% confidence interval

obtained using the standard error of each MR estimate. MR estimates were obtained from the MendelianRanomization R package.

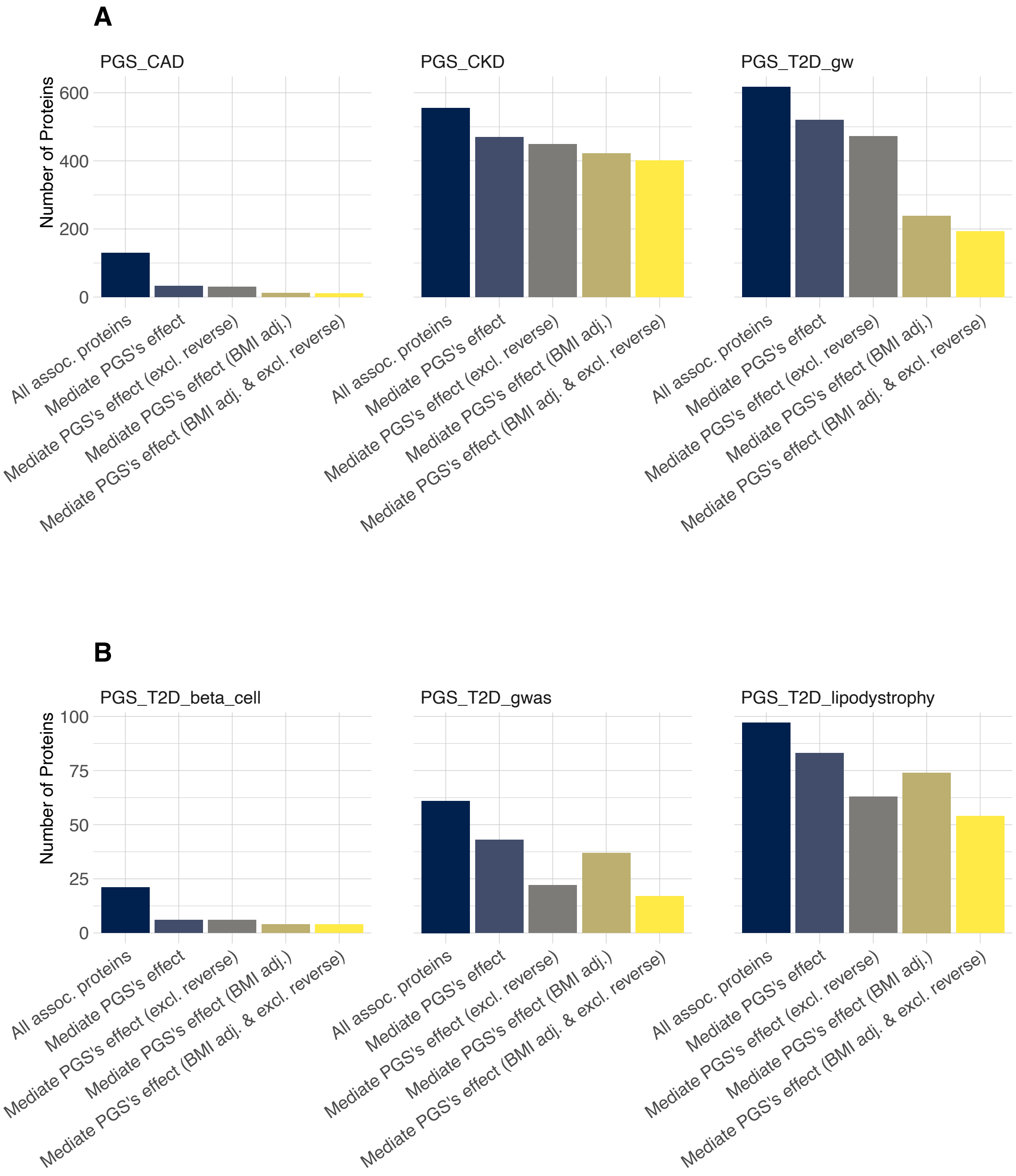

Supplementary Figure 18: Mediation analysis summary. From the left, the first column in each bar plot is the total number of proteins associated with a PGS, followed by the total number of proteins that significantly mediate the PGS's effect on incident disease (CKD, T2D, or CAD) the number of proteins that significantly mediate the PGS's effect on incident disease after excluding proteins found using reverse MR for the corresponding trait, the total number of proteins that significantly mediate the PGS's effect on incident disease after BMI adjustment, and the total number of proteins that significantly mediate the PGS's effect on incident disease after BMI adjustment and reverse MR exclusions. A: Results for cardiorenal PGS including PGS$_{T2D_gw}$. B: Results for GWAS-significant T2D PGS, including the

partitioned polygenic scores. Note that if a partitioned polygenic score was not shown, then it was not significantly associated with either incident T2D or not significantly associated with any proteins. For all panels, the sample size was 44,381 and mediation was performed using the medflex R package.

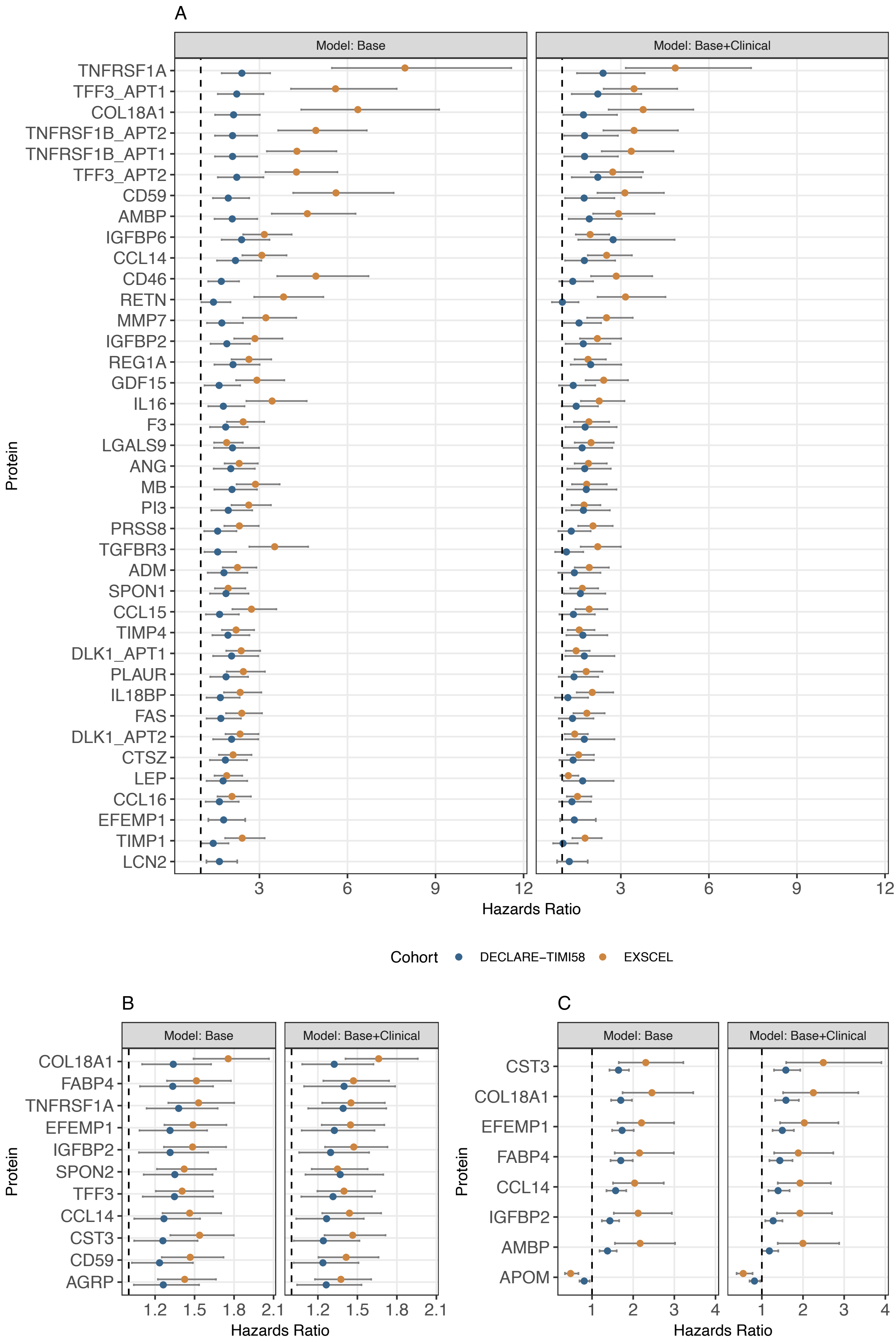
A
Model: Base
Model: Base+Clinical
TNFRSF1A
TFF3_APT1
COL18A1
TNFRSF1B_APT2
TNFRSF1B_APT1
TFF3_APT2
CD59
AMBP
IGFBP6
CCL14
CD46
RETN
MMP7
IGFBP2
REG1A
GDF15
IL16
F3
LGALS9
ANG
MB
PI3
PRSS8
TGFBR3
ADM
SPON1
CCL15
TIMP4
DLK1_APT1
PLAUR
IL18BP
FAS
DLK1_APT2
CTSZ
LEP
CCL16
EFEMP1
TIMP1
LCN2
Protein
3
6
9
12
Hazards Ratio
Cohort
DECLARE−TIMI58
EXSCEL
B
Model: Base
Model: Base+Clinical
COL18A1
FABP4
TNFRSF1A
EFEMP1
IGFBP2
SPON2
TFF3
CCL14
CST3
CD59
AGRP
Protein
1.2
1.5
1.8
2.1
Hazards Ratio
C
Model: Base
Model: Base+Clinical
CST3
COL18A1
EFEMP1
FABP4
CCL14
IGFBP2
AMBP
APOM
Protein
1
2
3
4
Hazards Ratio

Supplementary Figure 19: Cox regression in Randomized clinical trials (2nd time point). Results shown are from Cox proportional hazards regression of clinical trial outcomes in EXSCEL and DECLARE-TIMI58 using proteins measured in a second timepoint as the exposure (12 months for EXSCEL, 6 months for DECLARE). A. Renal outcome results, with EXSCEL labelled in yellow and DECLARE labelled in Blue. All proteins shown significantly replicated in DECLARE using the base model (basic covariates only, see Methods), shown on the left panel labelled "Model:Base". Panel on the right displays the results from a model that also includes clinical risk factors. B. Results for the major adverse cardiovascular event (MACE) outcomes in EXSCEL and DECLARE. C. Results for the hospitalization for heart failure (HHF) outcome in EXSCEL and DECLARE. Panels B and C are configured the same as panel A. For all panels, error bars represent the 95% confidence interval calculated using the standard error for each hazard ratio. For all panels, hazard rations were obtained using proportional hazards regression and the survival R package. EXSCEL had a sample size of 1,407 study participants from the placebo arm with available proteomics information, while DECLARE had a sample size of 497 study participants from the placebo arm with available proteomics information.

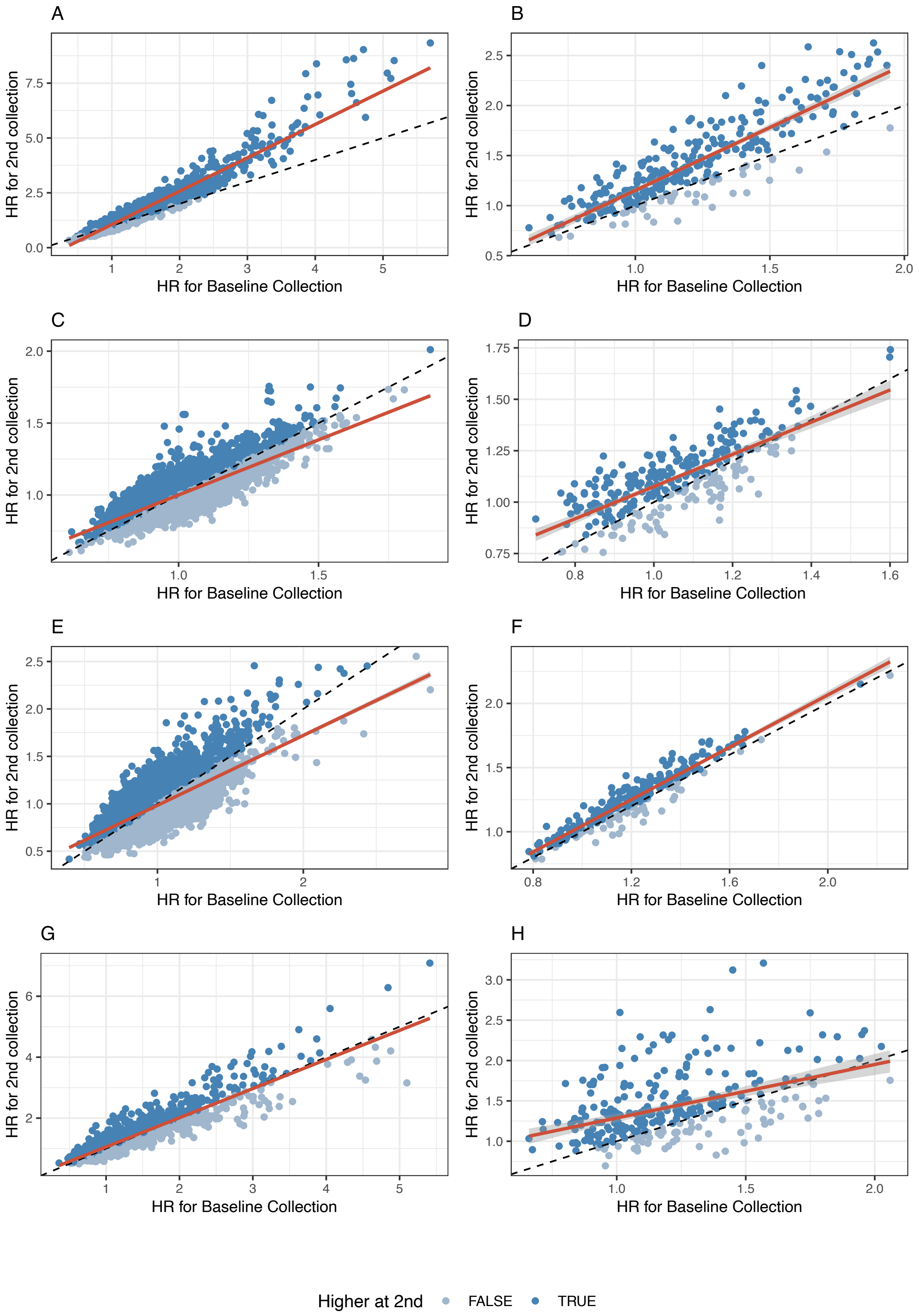
A
B
C
D
E
F
G
H
HR for 2nd collection
HR for Baseline Collection
Higher at 2nd
FALSE
TRUE

Supplementary Figure 20: Comparison of timepoints in Cox regression analysis. A. Comparison of hazard ratios (HR) using the baseline protein measurements (x-axis) and the 2nd timepoint measurements for the renal outcome in EXSCEL with the base model (excluding clinical covariates, see methods). B. Comparison of HR using the baseline protein measurements (x-axis) and the 2nd timepoint measurements for the renal outcome in DECLARE using the base model. C. Comparison of HR using the baseline protein measurements (x-axis) and the 2nd timepoint measurements for the major adverse cardiovascular event (MACE) outcome in EXSCEL with the base model. D. Comparison of HR using the baseline protein measurements (x-axis) and the 2nd timepoint measurements for the major adverse cardiovascular event (MACE) outcome in DECLARE with the base model. E. Comparison of HR using the baseline protein measurements (x-axis) and the 2nd timepoint measurements for the hospitalization for heart failure (HHF) outcome in EXSCEL with the base model. F. Comparison of HR using the baseline protein measurements (x-axis) and the 2nd timepoint measurements for the HHF outcome in DECLARE with the base model. G. Comparison of HR using the baseline protein measurements (x-axis) and the 2nd timepoint measurements for the renal outcome in EXSCEL, with the 2nd timepoint model adjusted for the baseline protein measurement. H. Comparison of HR using the baseline protein measurements (x-axis) and the 2nd timepoint measurements for the renal outcome in DECLARE, with the 2nd timepoint model adjusted for the baseline protein measurement. In all panels, the blue colour corresponds to proteins that had a larger HR at the 2nd timepoint. EXSCEL had a sample size was 1,407 study participants from the placebo arm with available proteomics information, while DECLARE had a sample size of 497 study participants from the placebo arm with available proteomics information.

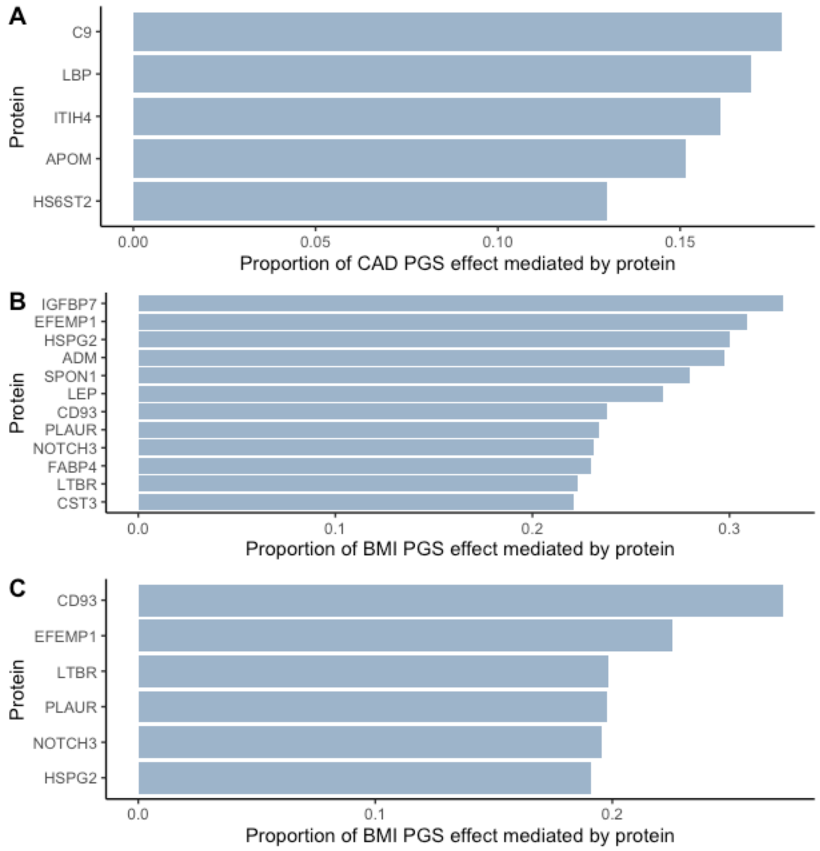

Supplementary Figure 21: Mediation analysis in EXSCEL and DECLARE. The mediation framework modelled the PGS as the direct effect, the protein as the indirect (mediating) effect, and the total effect (PGS + protein) using the medflex R package. The X-axis is the proportion of the PGS effect mediated by protein, defined as indirect effect (protein) / total effect (PGS + protein). A. Mediation for CAD PGS and the MACE outcome in EXSCEL. B. Mediation for the BMI PGS and the hospitalization for heart failure endpoint. C. Mediation for the BMI PGS and the hospitalization for heart failure endpoint after adjusting for baseline BMI.

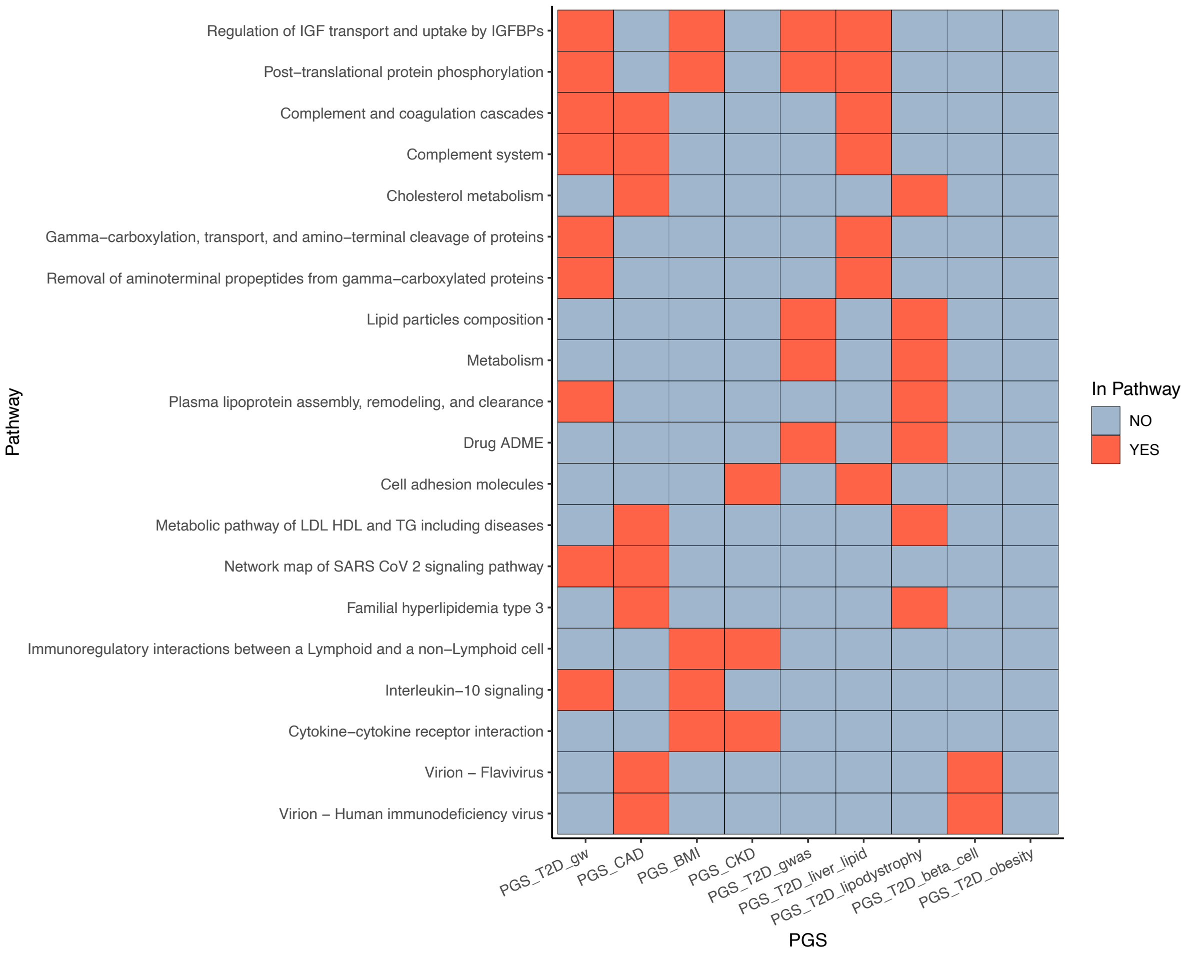

Supplementary Figure 22: Pathway enrichment. KEGG, REACTOME, and WIKIPATH pathways significantly enriched in the PGS-associated proteins. Red squares indicate if that pathway (y-axis) was also significantly enriched in the proteins associated with the given score (x-axis). Note that pathways enriched in two or more PGS-protein sets are shown here. A further 75 pathways were only enriched in one set, such as TNFs bind their physiological receptors for the PGS_{CKD}.

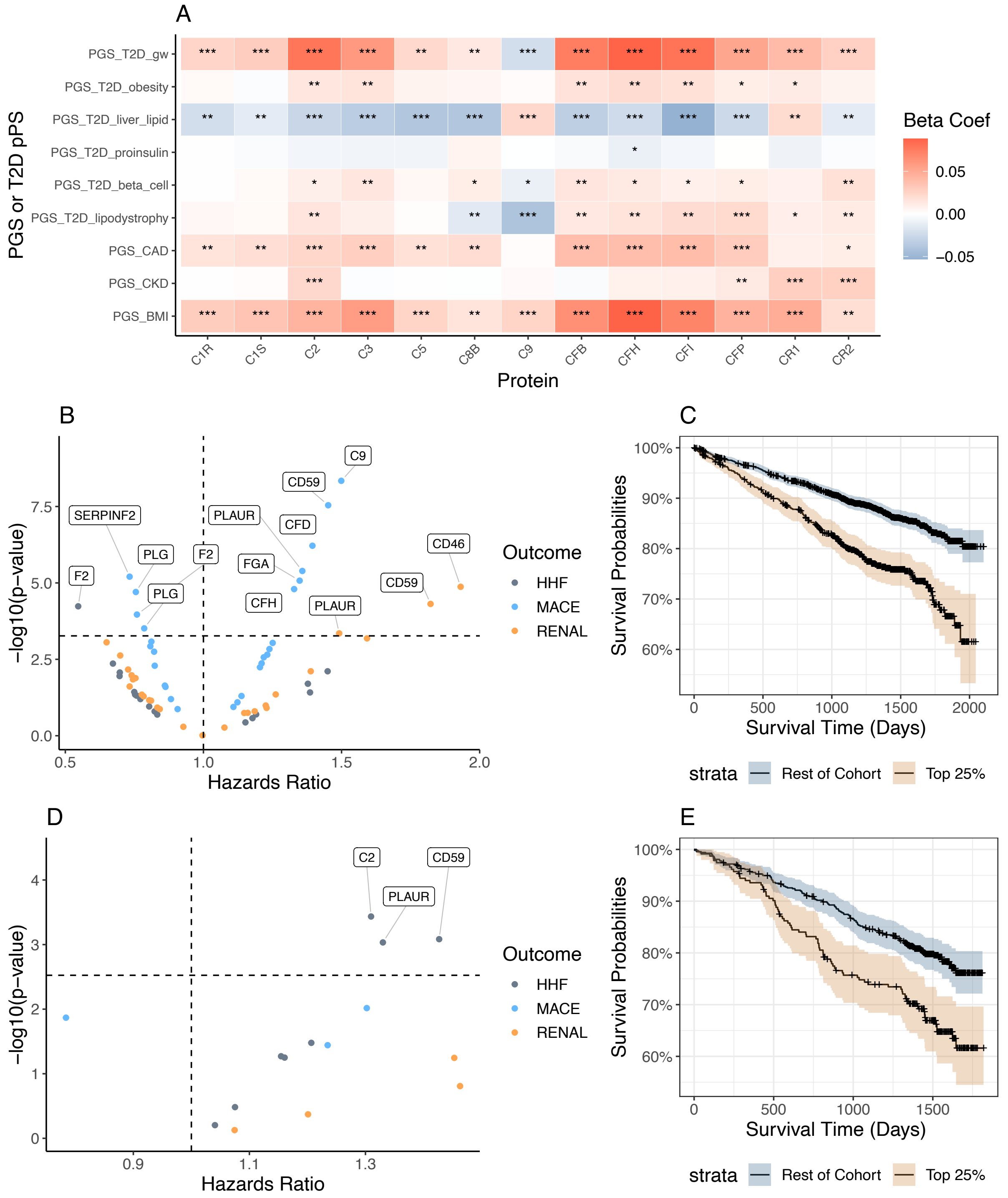

Figure 23: Complement and coagulation cascade pathway highlights. A: PGS associations with complement-related proteins. A single asterisk (*) indicates the association was nominally significant, while two (**) indicates significance using FDR and three (***) indicates significance using a Bonferroni correction. B: Associations of proteins (measured at baseline) in this pathway with clinical trial outcomes in EXSCEL using Cox regression. C. Kaplan Meier curve demonstrating the impact of CD59 levels on the composite cardiovascular endpoint in EXSCEL. D: Associations of proteins (measured at baseline) in this pathway with clinical trial outcomes in DECLARE using Cox regression. E. Kaplan Meier curve demonstrating the impact of CD59 levels on the composite cardiovascular endpoint in

DECLARE. For panels B and D, the dashed line corresponds the p-value threshold where FDR < 5% (when applied to the proteins in this pathway). Note that panels B and D display unadjusted, two-sided p-values obtained from Cox proportional hazards regression.
