# Supplementary material for: Identification of plasma proteomic markers underlying polygenic risk of type 2 diabetes and related comorbidities
Source: Nat Commun. 2025 Mar 3;16:2124. doi: 10.1038/s41467-025-56695-z (PMC11876343; doi:10.1038/s41467-025-56695-z)
Supplement: Supplementary file 2 — Description of Additional Supplementary Files [file 41467_2025_56695_MOESM2_ESM.pdf]

Description of Additional Supplementary Files

File Name: Supplementary Data 1

Description: Description of cohorts used in this study

File Name: Supplementary Data 2

Description: Description of UKB phenotype definitions used in this study

File Name: Supplementary Data 3

Description: Description of phenotypes used in this study for EXSCEL and DECLARE-TIMI58

File Name: Supplementary Data 4

Description: Description of external data used for polygenic score construction

File Name: Supplementary Data 5

Description: Description of polygenic scores used in this study

File Name: Supplementary Data 6

Description: Results of PGS-protein association analyses in UKB-PPP

File Name: Supplementary Data 7

Description: Results of PGS-protein association analyses in UKB-PPP; also includes mediation results for PGS_T2D_gw, BMI, and proteins

File Name: Supplementary Data 8

Description: Summary of proteins associated with the PGS_T2D_gw and another score with opposite directions of effect

File Name: Supplementary Data 9

Description: Results of 2-sample MR analysis in UKB-PPP, including colocalization evidence

File Name: Supplementary Data 10

Description: Results of 2-sample analysis using MR-Link2

File Name: Supplementary Data 11

Description: Results of multi-variable MR analysis with BMI and protein as exposure and T2D as the outcome

File Name: Supplementary Data 12

Description: Results of UKB-PPP mediation analysis

File Name: Supplementary Data 13

Description: Results of 2-sample reverse MR analysis in UKB-PPP

File Name: Supplementary Data 14

Description: Results of survival analysis for trial outcomes in EXSCEL using proteins as exposure

File Name: Supplementary Data 15

Description: Results of survival analysis for trial outcomes in DECLARE using proteins as exposure

File Name: Supplementary Data 16

Description: Results of survival analysis for trial outcomes in DECLARE and EXSCEL using PGS as exposure

File Name: Supplementary Data 17

Description: Results of PGS-protein association analyses in DECLARE

File Name: Supplementary Data 18

Description: Results of PGS-protein association analyses in EXSCEL

File Name: Supplementary Data 19

Description: Results of EXSCEL mediation analysis

File Name: Supplementary Data 20

Description: Results of DECLARE mediation analysis

File Name: Supplementary Data 21

Description: Summary of proteins with causal evidence in manuscript, along with external evidence
