# Supplementary material for: Identification of plasma proteomic markers underlying polygenic risk of type 2 diabetes and related comorbidities
Source: Nat Commun. 2025 Mar 3;16:2124. doi: 10.1038/s41467-025-56695-z (PMC11876343; doi:10.1038/s41467-025-56695-z)
Supplement: Supplementary file 4 — Reporting Summary [file 41467_2025_56695_MOESM4_ESM.pdf]

nature portfolio

Corresponding author(s): Douglas Loesch, Dirk Paul

Last updated by author(s): Dec 5, 2024

Reporting Summary

Nature Portfolio wishes to improve the reproducibility of the work that we publish. This form provides structure for consistency and transparency in reporting. For further information on Nature Portfolio policies, see our Editorial Policies and the Editorial Policy Checklist.

Statistics

For all statistical analyses, confirm that the following items are present in the figure legend, table legend, main text, or Methods section.

n/a	Confirmed	
☐	☒	The exact sample size (*n*) for each experimental group/condition, given as a discrete number and unit of measurement
☐	☒	A statement on whether measurements were taken from distinct samples or whether the same sample was measured repeatedly
☐	☒	The statistical test(s) used AND whether they are one- or two-sided<br>*Only common tests should be described solely by name; describe more complex techniques in the Methods section.*
☐	☒	A description of all covariates tested
☐	☒	A description of any assumptions or corrections, such as tests of normality and adjustment for multiple comparisons
☐	☒	A full description of the statistical parameters including central tendency (e.g. means) or other basic estimates (e.g. regression coefficient) AND variation (e.g. standard deviation) or associated estimates of uncertainty (e.g. confidence intervals)
☐	☒	For null hypothesis testing, the test statistic (e.g. *F*, *t*, *r*) with confidence intervals, effect sizes, degrees of freedom and *P* value noted<br>*Give P values as exact values whenever suitable.*
☐	☒	For Bayesian analysis, information on the choice of priors and Markov chain Monte Carlo settings
☒	☐	For hierarchical and complex designs, identification of the appropriate level for tests and full reporting of outcomes
☐	☒	Estimates of effect sizes (e.g. Cohen's *d*, Pearson's *r*), indicating how they were calculated

Our web collection on statistics for biologists contains articles on many of the points above.

Software and code

Policy information about availability of computer code

Data collection	No software was used for data collection.
Data analysis	This study was carried out using only publicly available software. Data processing, regression, and survival analyses were done using R 4.0.2 and the R packages stats and survival (https://github.com/therneau/survival). Causal inference analyses were performed using R 4.2.2 and the R packages medflex v0.6-10 (https://github.com/jmpsteen/medflex), MendelianRandomization v0.7.0 (https://cran.r-project.org/web/packages/MendelianRandomization/index.html), MVMR v0.4 (https://github.com/WSpiller/MVMR/tree/master), mediation v4.5.0 (https://cran.r-project.org/web/packages/mediation/index.html), and coloc v5.1.0.1 (https://github.com/chr1swallace/coloc), plus the MR-Link-2 v.0.1.2 Python package (https://github.com/adriaan-vd-graaf/mrlink2). Genetic analyses and genotype quality control was performed using PLINK v1.90b6.18 (https://www.cog-genomics.org/plink/2.0/) and PLINK v2.00a4LM (https://www.cog-genomics.org/plink/2.0/). Unless otherwise specified, genotype imputation was performed using EAGLE 2.4.1 (https://alkesgroup.broadinstitute.org/Eagle/) and BEAGLE 4.1 (https://faculty.washington.edu/browning/beagle/b4_1.html). Polygenic scores were trained using PRS-CS (https://github.com/getian107/PRScs) and PRS-CSx (https://github.com/getian107/PRScsx). GWAS data for MR and colocalization was generated using REGENIE v3.1.2 (https://github.com/rgcgithub/regenie). Pathway analyses was performed using the g:Profiler web portal (https://biit.cs.ut.ee/gprofiler/gost). Analysis scripts are available at https://github.com/astrazeneca-cgr-publications/plasma-proteomic-markers-prs-t2d-scripts.

For manuscripts utilizing custom algorithms or software that are central to the research but not yet described in published literature, software must be made available to editors and reviewers. We strongly encourage code deposition in a community repository (e.g. GitHub). See the Nature Portfolio guidelines for submitting code & software for further information.

Data

Policy information about availability of data

All manuscripts must include a data availability statement. This statement should provide the following information, where applicable:

- Accession codes, unique identifiers, or web links for publicly available datasets
- A description of any restrictions on data availability
- For clinical datasets or third party data, please ensure that the statement adheres to our policy

All results (filtered for a nominal p-value of < 0.05) are available in the supplementary materials, many of which are also available via the web portal. The web portal allows download of the underlying data. Requests to access to UK Biobank data can be made here: https://www.ukbiobank.ac.uk/enable-your-research/apply-for-access. Clinical trial data can be accessed following AstraZeneca's data sharing policies: https://www.astrazenecaclinicaltrials.com/our-transparency-commitments/. All PGS used in the study will be deposited at the PGS Catalog (https://www.pgscatalog.org/).

Research involving human participants, their data, or biological material

Policy information about studies with human participants or human data. See also policy information about sex, gender (identity/presentation), and sexual orientation and race, ethnicity and racism.

Reporting on sex and gender	Self-reported sex information was collected by the UK Biobank, EXSCEL, and DECLARE-TIMI 58 study groups. Sex was included a covariate in regression models. We did not perform any sex-specific analyses as this is out of scope for this project.
Reporting on race, ethnicity, or other socially relevant groupings	This study made use of genetically-predicted ancestry labels to reduce confounding and examine the cross-ancestry portability of our analysis results as this is an ongoing concern for polygenic scores. These labels were generated using the KING software package and the 1000 Genomes Project participants as genetic references. KING first performs principal component analysis on the 1000 Genomes data, then projects the target data into the same principal component space. Finally, KING uses a support vector machine, the principal components, and 1000 Genomes-provided super population labels to assign ancestry labels to the target data. We then retained ancestry labels if they had a posterior probability greater than 0.9. In regression analyses, we also adjusted for genetic principal components to control for population stratification.
Population characteristics	The UK Biobank participants have a mean age of 56.5 years and are 54% female. The UK Biobank subset with proteomics data is largely similar, with a mean age of 56.8 years and are 54% female. The UK Biobank is a population-level biobank and thus was not recruited for any particular indication. The EXSCEL cohort has a mean age of 62.4 years and was 38% female. The DECLARE-TIMI 58 cohort has a mean age of 64.0 years and are 36% female. For both EXSCEL and DECLARE-TIMI 58, study participants had type 2 diabetes and cardiovascular risk factors. All subjects in this study were genotyped, while a subset had their plasma proteomes profiled.
Recruitment	We did not perform any recruiting for this study. The UK Biobank recruited participants, and potential biases there are well-documented (relatively healthy, majority European ancestry, narrow age band). The clinical trials also managed their own recruitment. In both trials, patients needed to be diagnosed with type 2 diabetes and have cardiovascular risk factors. Note that the clinical trials are on average older than the UK Biobank, as well as more diverse.
Ethics oversight	The UK Biobank operates with an approval from the North West Multi-centre Research Ethics Committee (MREC) as a Research Tissue Bank (RTB). All UK Biobank participants provided consent. For the clinical trials (EXSCEL and DECLARE-TIMI 58), all participants provided written informed consent, and the trial protocol was approved by ethics committees at each of the trial's participating sites.

Note that full information on the approval of the study protocol must also be provided in the manuscript.

Field-specific reporting

Please select the one below that is the best fit for your research. If you are not sure, read the appropriate sections before making your selection.

☒ Life sciences ☐ Behavioural & social sciences ☐ Ecological, evolutionary & environmental sciences

For a reference copy of the document with all sections, see nature.com/documents/nr-reporting-summary-flat.pdf

Life sciences study design

All studies must disclose on these points even when the disclosure is negative.

Sample size	Sample sizes were dictated by the cohorts used for this study as we did not perform any primary data collection. We used 492,520 samples from the UK Biobank, 54,781 of which had proteomic information. We used 12,814 samples from DECLARE-TIMI, 915 of which had proteomic information. Finally, we used 4,666 samples from EXSCEL, 2823 of which had proteomic information.
Data exclusions	We excluded close relatives defined as having a 2nd-degree relationship using a kinship matrix estimated by the KING software package. In many analyses in the UK Biobank, we also excluded subjects who had prevalent cardiometabolic disease at the time of data collection. Both of these exclusions were done to avoid over-fitting and potential collider bias.
Replication	For the polygenic score associations with circulating proteins, we used the same internal replication strategy employed by Sun et al. 2023, the

Replication	flagship manuscript from the UK Biobank proteomics consortium. We only discuss proteins that passed internal replication in our manuscript. In our analyses with the clinical trials, we used one trial for discovery and the second trial for replication. Note that in this case, only a relatively small subset of proteins were available in both trials.
Randomization	Our study was non-interventional, the clinical trial data was only used in a post-hoc fashion irrespective of treatment arm.
Blinding	Our study was non-interventional, the clinical trial data was only used in a post-hoc fashion irrespective of treatment arm.

Reporting for specific materials, systems and methods

We require information from authors about some types of materials, experimental systems and methods used in many studies. Here, indicate whether each material, system or method listed is relevant to your study. If you are not sure if a list item applies to your research, read the appropriate section before selecting a response.

Materials & experimental systems

n/a	Involved in the study
☒	☐ Antibodies
☒	☐ Eukaryotic cell lines
☒	☐ Palaeontology and archaeology
☒	☐ Animals and other organisms
☐	☒ Clinical data
☒	☐ Dual use research of concern
☒	☐ Plants

Methods

n/a	Involved in the study
☒	☐ ChIP-seq
☒	☐ Flow cytometry
☒	☐ MRI-based neuroimaging

Clinical data

Policy information about clinical studies

All manuscripts should comply with the ICMJE guidelines for publication of clinical research and a completed CONSORT checklist must be included with all submissions.

Clinical trial registration	EXSCEL's registration number on ClinicalTrials.gov is NCT01144338. DECLARE-TIMI 58's registration number is NCT01730534.
Study protocol	The study protocols for both DECLARE-TIMI 58 and EXSCEL are available on NEJM.org
Data collection	Our manuscript involved a post-hoc analysis of clinical trials. As such, we did not perform any data collection. This was performed by the groups who performed the trials. Information is available in the associated publications for the trials. (DOI for DECLARE: 10.1056/NEJMoa1812389; DOI for EXSCEL: 10.1056/NEJMoa1612917)
Outcomes	Our manuscript involved a post-hoc analysis of clinical trials. As such, we did not define any outcomes. These were defined by the teams that ran the clinical trials. Information is available via the study protocols and the associated publications. (DOI for DECLARE: 10.1056/NEJMoa1812389; DOI for EXSCEL: 10.1056/NEJMoa1612917)

Plants

Seed stocks	*Report on the source of all seed stocks or other plant material used. If applicable, state the seed stock centre and catalogue number. If plant specimens were collected from the field, describe the collection location, date and sampling procedures.*
Novel plant genotypes	*Describe the methods by which all novel plant genotypes were produced. This includes those generated by transgenic approaches, gene editing, chemical/radiation-based mutagenesis and hybridization. For transgenic lines, describe the transformation method, the number of independent lines analyzed and the generation upon which experiments were performed. For gene-edited lines, describe the editor used, the endogenous sequence targeted for editing, the targeting guide RNA sequence (if applicable) and how the editor was applied.*
Authentication	*Describe any authentication procedures for each seed stock used or novel genotype generated. Describe any experiments used to assess the effect of a mutation and, where applicable, how potential secondary effects (e.g. second site T-DNA insertions, mosiacism, off-target gene editing) were examined.*
